# Dynamic photosynthetic labeling and carbon-positional mass spectrometry monitor in vivo RUBISCO carbon assimilation rates

**DOI:** 10.1093/plphys/kiaf020

**Published:** 2025-01-21

**Authors:** Yogeswari Rajarathinam, Luisa Wittemeier, Kirstin Gutekunst, Martin Hagemann, Joachim Kopka

**Affiliations:** Max-Planck-Institute of Molecular Plant Physiology, Am Mühlenberg 1, D-14476 Potsdam-Golm, Germany; Environmental and Biochemical Sciences, The James Hutton Institute, Invergowrie, Dundee DD2 5DA, UK; Max-Planck-Institute of Molecular Plant Physiology, Am Mühlenberg 1, D-14476 Potsdam-Golm, Germany; Molecular Plant Physiology, Bioenergetics in Photoautotrophs, University Kassel, Heinrich-Plett-Straße 40, D-34132 Kassel, Germany; Plant Physiology Department, Institute of Biological Sciences, Rostock University, Albert-Einstein-Straße 3, D-18059 Rostock, Germany; Max-Planck-Institute of Molecular Plant Physiology, Am Mühlenberg 1, D-14476 Potsdam-Golm, Germany

## Abstract

RIBULOSE-1,5-BISPHOSPHATE CARBOXYLASE/OXYGENASE (RUBISCO) is the most abundant enzyme and CO_2_ bio-sequestration system on Earth. Its in vivo activity is usually determined by ^14^CO_2_ incorporation into 3-phosphoglycerate (3PGA). However, the radiometric analysis of 3PGA does not distinguish carbon positions. Hence, RUBISCO activity that fixes carbon into the 1-C position of 3PGA and Calvin–Benson–Bassham (CBB) cycle activities that redistribute carbon into its 2-C and 3-C positions are not resolved. This study aims to develop technology that differentiates between these activities. In source fragmentation of gas chromatography-mass spectrometry (GC-MS) enables paired isotopologue distribution analyses of fragmented substructures and the complete metabolite structure. GC-MS measurements after dynamic photosynthetic ^13^CO_2_ labeling allowed quantification of the ^13^C fractional enrichment (E*^13^C*) and molar carbon assimilation rates (A*^13^C*) at carbon position 1-C of 3PGA by combining E*^13^C* from carbon positions 2,3-C_2_ and 1,2,3-C_3_ with quantification of 3PGA concentrations. We validated the procedure using two GC-time of flight-MS instruments, operated at nominal or high mass resolution, and tested the expected 3PGA positional labeling by in vivo glycolysis of positional labeled glucose isotopomers. Mutant analysis of the highly divergent GLYCERALDEHYDE-3-PHOSPHATE DEHYDROGENASEs (GAPDH1 and 2) from *Synechocystis* sp. PCC 6803 revealed full inactivation of the CBB cycle with maintained RUBISCO activity in *Δgapdh2* and a CBB cycle modulating role of GAPDH1 under fluctuating CO_2_ supply. RUBISCO activity in the CBB-deficient *Δgapdh2* can re-assimilate CO_2_ released by catabolic pathways. We suggest that RUBISCO activity in *Synechocystis* can scavenge carbon lost through the pentose phosphate pathway or other cellular decarboxylation reactions.

## Teaser

An integrative approach enables the analysis of RUBISCO-mediated carbon assimilation and reveals continued RUBISCO activity in cyanobacteria following inactivation of the Calvin–Benson–Bassham cycle.

## Introduction

RIBULOSE-1,5-BISPHOSPHATE CARBOXYLASE/OXYGENASE (RUBISCO) is the essential part of the Calvin–Benson–Bassham (CBB) cycle (Prywes et al. 2023). Evolution of RUBISCO and the CBB cycle allows cyanobacteria, algae, and land plants to produce the photosynthetic biomass that sustains life on earth (Erb and Zarzycki 2018). RUBISCO (EC 4.1.1.39) catalyzes CO_2_ fixation through carboxylation of ribulose-1,5-bisphosphate (RuBP) and subsequent cleavage into two 3-phosphoglycerate (3PGA) molecules (Bassham et al. 1954; Lorimer 1981; Sharkey 2023). The enzyme reacts competitively with O_2_ resulting in 3PGA and 2-phosphoglycolate (2PG). 2PG inhibits essential cellular enzymes and needs detoxification by photorespiration at the expense of energy and loss of assimilated carbon (Walker et al. 2016, 2024). RUBISCO is present in all domains of life. It evolved early in earth’s history likely from an enolase involved in methionine salvage (Ashida et al. 2003). RUBISCO likely had a heterotrophic CO_2_ scavenging function as evidenced by archaeal nucleoside degrading carbon metabolism (Aono et al. 2015) before it acquired its photoautotrophic role (Schönheit et al. 2016; Erb and Zarzycki 2018). These steps took place before the first oxygenation event, when atmospheric CO_2_ concentrations were high and O_2_ low. Hence, RUBISCO evolution became trapped in a tradeoff between optimizing enzyme activity and CO_2_ specificity (Prywes et al. 2023). This impasse is still the grand obstacle to modern synthetic biology (Erb and Zarzycki 2018) that aims to optimize RUBISCO performance for improved crop production and bio-sequestration of atmospheric CO_2_ (Gutteridge and Pierce 2006).

Measurement of RUBISCO activity in vivo has been the grand challenge in photosynthesis research and was instrumental for the discovery of the CBB cycle (Sharkey 2023). Dynamic photosynthetic ^14^CO_2_ labeling proved that 3PGA is the first assimilation product (Bassham et al. 1950, 1954; Calvin 1956, 1962). Carbon bond specific chemical cleavage and monitoring of the reaction products demonstrated that 1-C of 3PGA receives the radio-labeled ^14^C-atom and unraveled the RuBP regenerating aldolase, transaldolase and transketolase reactions of the CBB cycle. These reactions concomitantly rearrange the carbon constitution of the CBB cycle’s carbohydrate intermediates. Photosynthetic carbon assimilation can be monitored by CO_2_ gas exchange analyses (von Caemmerer and Farquhar 1981; Long and Bernacchi 2003; von Caemmerer 2020) or ^14^C incorporation into biomass, e.g. (Farrar 1993). These technologies do not directly distinguish between the alternative carbon assimilation routes through RUBISCO or PHOSPHOENOLPYRUVATE CARBOXYLASE. RUBISCO activity is specifically and reliably measured by incorporation of ^14^CO_2_ into 3PGA and radiometry (Lorimer et al. 1977; Parry et al. 1997; Kubien et al. 2011) or by spectrophotometric assays (Racker 1962; Ward and Keys 1989; Sulpice et al. 2007; Sales et al. 2020). Spectrophotometric assays measure orthogonal in vitro activities of activated or nonactivated RUBISCO preparations from photosynthetic tissues and are typically consistent with the radiometric assay but may underestimate (Sales et al. 2020). Likewise, in vivo estimates of RUBISCO activity by gas exchange and in vitro measurements may not agree, e.g. (Rogers et al. 2001). RNA-sensor based fluorometric assays are a recent addition to the tool box of RUBISCO assays (Faisal et al. 2024) that are available to characterize RUBISCO modifications through synthetic biology or to validate experimentally the predictions made by metabolic modeling.

Measurements by photosynthetic labeling and incorporation of ^14^CO_2_ into 3PGA report the in vivo status of RUBISCO activity and reflect effects of cellular enzyme amount, activation status, availability of the substrates in the vicinity of the enzyme, and metabolic regulation by effectors (Prywes et al. 2023; Sharkey 2023). Radiometry of 3PGA does not distinguish between its carbon positions. Consequently, RUBISCO activity that fixes carbon into 1-C position of 3PGA is not differentiated from CBB cycle activities that redistribute assimilated carbon to 2-C and 3-C of 3PGA. If RUBISCO activity limits carbon assimilation and the CBB cycle has a faster rate than RUBISCO, this analytical limitation can be negligible. All three carbon atoms of 3PGA will be labeled homogenously and at equal rates. However, in physiological states that are not limited by RUBISCO, 1-C of 3PGA should be labeled faster and the rate of label-redistribution within 3PGA should lag behind. Providing technology that differentiates between RUBISCO and CBB cycle activities motivates this study.

Gas chromatography coupled to mass spectrometry (GC-MS) combined with chemical derivatization methods, such as trimethylsilylation, that make nonvolatile compounds volatile, is a routine technology for the profiling of primary metabolism (Fiehn et al. 2000; Lisec et al. 2006). Compounds that are separated by GC are subsequently ionized to become detectably by mass spectrometry. The high ionization energy, typically 70 eV, causes compound fragmentation within the ion source. Such *in source* fragmentation reactions have been proposed and applied to carbon-positional analyses of primary metabolites, such as organic acids of the tricarboxylic acid (TCA) cycle (Okahashi et al. 2019), aspartate (Wittemeier et al. 2024), or glutamate (Lima et al. 2021). These fragmentation reactions can replace in-line, the laborious chemical cleavage reactions that led to the unraveling of the CBB cycle (Calvin 1962). In this study, we measure carbon assimilation into 1-C position of 3PGA by *in source* fragmentation that is integral to GC-time of flight (TOF)-MS (GC-(TOF)-MS). We explore two ionization technologies, the highly fragmenting electron impact ionization (EI) of gas chromatography-electron impact ionization-mass spectrum (GC-EI-(TOF)MS) operated at nominal mass resolution and less fragmenting atmospheric pressure chemical ionization (APCI) of gas chromatography-atmospheric pressure chemical ionization-high resolution mass spectrum (GC-APCI-(TOF)MS) with high mass resolution. We combine the widely applied and easy to transfer GC-MS based metabolite profiling technology with dynamic ^13^CO_2_ pulse labeling instead of radioactively labeled ^14^CO_2_ (Bassham et al. 1950, 1954; Calvin 1956, 1962). We carefully explore analytical aspects, such as carbon-position specificity and interferences by MS instrument bias or coeluting isobaric compounds. Compounds of equal mass to charge ratio (m/z) are frequent in the typically highly complex metabolite preparations of metabolomic studies and may interfere. We combine the optimized results from both GC-(TOF)MS technologies to determine ^13^C fractional enrichment (E*^13^C*) and the molar concentrations of ^13^C within 3PGA (C*^13^C*) to account for concentration changes of 3PGA that can occur during dynamic pulse labeling and must be expected when different metabolic states are compared. Together, these data allow calculations of positional molar ^13^C assimilation rates (A*^13^C*) into 3PGA.

For a first application, we chose the cyanobacterium *Synechocystis sp.* PCC 6803 (in the following: *Synechocystis*) as an easy to cultivate and phylogenetically ancient photosynthetic model organism that can be highly labeled by photosynthetic ^13^CO_2_ uptake, e.g. (Huege et al. 2011). *Synechocystis* belongs to the β-cyanobacteria and has a class IB RUBISCO, like green algae and plants (Rae et al. 2013; Kerfeld and Melnicki 2016). RUBISCO of *Synechocystis* wild type (WT) has a catalytic activity K_cat_ of 500 to 1000 min^−1^ and a Michaelis constant K_m_ (RuBP) of ∼ 140 µM, quantified by different studies using ^14^CO_2_ activity assays (Marcus et al. 2005, 2011). When acclimated to low inorganic carbon (Ci) availability of the current ambient atmospheric CO_2_, RUBISCO is assembled into β-carboxysomes. These are highly structured protein microbodies that serve as part of a CO_2_-concentrating mechanism (CCM) and act together with activated uptake of Ci (bicarbonate and CO_2_) (Price et al. 2008; Orf et al. 2015; Hagemann et al. 2021) and increase CO_2_ concentrations locally in the vicinity of RUBISCO (Kerfeld and Melnicki 2016). At high CO_2_ concentrations that prevailed early in earth’s history when cyanobacteria evolved or are used for modern biotechnological applications, the CCM is thought to be largely inactive (McGinn et al. 2003; Woodger et al. 2003). To test our technology, we probe *Synechocystis* cells that were preacclimated to high CO_2_ with a high ^13^CO_2_ pulse. Next to this steady-state condition, we include a nonsteady-state setup of cells that are preacclimated to low CO_2_ and probed by a high ^13^CO_2_ pulse. In both cases we expect that RUBISCO is nonlimiting for carbon assimilation but 3PGA concentrations of the differently preacclimated cells are known to differ (Orf et al. 2015).

GLYCERALDEHYDE-3-PHOSPHATE DEHYDROGENASE (GAPDH) catalyzes a central metabolic control step of the *Synechocystis* CBB cycle as well as glycolysis (Lucius and Hagemann 2024). GAPDH2 of *Synechocystis* has dual cosubstrate specificity, uses NAD as well as NADP, and is thought to take part in the anabolic carbon-flow of the CBB cycle (Lucius et al. 2022; Schulze et al. 2022). GAPDH1 is NAD-specific, nonessential, and likely has a catabolic function in glycolytic processes (Koksharova et al. 1998). We investigate the role of these two highly divergent GAPDH enzymes (Figge et al. 1999) by analyzing the previously generated and characterized *Δgapdh1* and *Δgapdh2* mutants in comparison to the *Synechocystis* WT. Our technology allows us to propose a role of GAPDH1, which has long been an enigma and we prove in vivo that the GAPDH1 enzyme in the nonphotoautotrophic *Δgapdh2* mutant does not support a CBB cycle. Surprisingly, we demonstrate RUBISCO activity in the *Δgapdh2* mutant and find evidence of a third carbohydrate-metabolizing pathway in *Synechocystis* next to the glycolytic Embden–Meyerhof–Parnas (EMP) and the oxidative pentose phosphate (OPP) pathways (Makowka et al. 2020). We propose that this path uses RUBISCO for the re-assimilation of CO_2_ that is lost through decarboxylation during the oxidative phase of the OPP path and other catabolic decarboxylation reactions. Hence, this finding supports the possible ancient role of RUBISCO as catabolic CO_2_ scavenger (Aono et al. 2015; Schönheit et al. 2016; Erb and Zarzycki 2018).

## Results

### Experimentally validated in silico fragmentation analyses predict carbon-positional monitoring options of 3-phosphoglyceric acid

**Figure 1.**
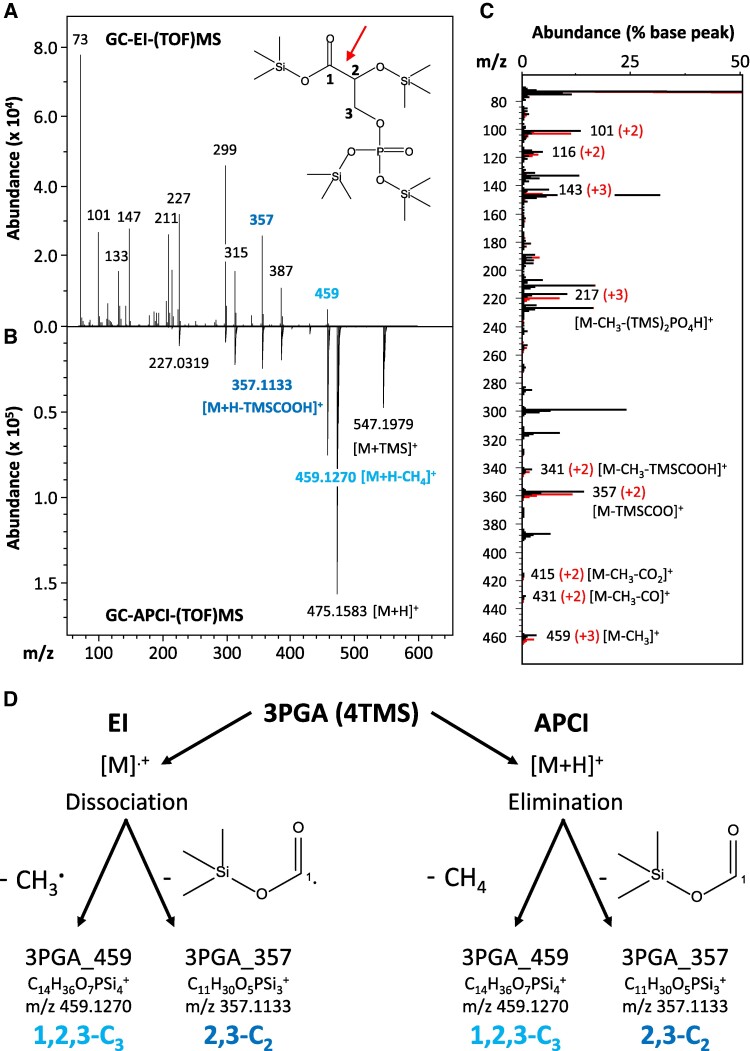
Mass spectral analysis of pertrimethylsilylated 3-phosphoglyceric acid, 3PGA (4TMS), by two independent GC-(TOF)MS technologies. **A)** GC-EI-(TOF)MS. The inserted molecular structure has chemical formula C_15_H_39_O_7_PSi_4_, and exact monoisotopic mass 474.1511. Numbers in the structure indicate carbon atom positions. **B)** Inverted display of an aligned GC-APCI-(TOF)MS. Nominal masses (GC-EI-(TOF)MS) and exact masses (GC-APCI-(TOF)MS) of abundant (arbitrary units) mass fragments and molecular adduct-ions are indicated. Two fragment ions used in this study are highlighted in light and dark blue. TMS represents a -Si(CH_3_)_3_ moiety, m/z (mass to charge ratio). **C)** Comparison of a completely in vivo ^13^C-labeled GC-EI mass spectrum (red) to an overlay of a nonlabeled, ambient GC-EI mass spectrum (black) of 3PGA (4TMS). Both mass spectra are scaled to the nonlabeled base peak, m/z 73 (% base peak). Fragments with ^13^C-induced mass shifts are annotated, e.g. +2 or +3 amu (red numbers in brackets). **D)** In silico fragmentation analysis of 3PGA (4TMS). Predicted fragmentation reactions of a molecular radical cation [M]^+^ that is generated by EI and of a proton adduct, [M + H]^+^, generated by APCI. The mechanisms of *in source* fragmentation differ between GC-EI-(TOF)MS and GC-APCI-(TOF)MS. Dissociative reactions of EI release noncharged radicals from [M]^.+^. APCI generated proton adducts, [M + H]^+^, are subject to neutral eliminations. Both technologies generate abundant mass fragments of nominal mass to charge ratios (m/z) 459 and 357 amu. These fragments contain either all carbon atoms, i.e. 1,2,3-C_3_, or the two carbon atoms, 2,3-C_2_ of 3PGA. **A)** and **D)** Carbon atom positions 1-C, 2-C, and 3-C of 3PGA are indicated. **A)** The red arrow marks the common cleavage site of GC-EI-(TOF)MS and GC-APCI-(TOF)MS between 1-C and 2-C of 3PGA.

GC-EI-(TOF)MS and GC-APCI-(TOF)MS analyses generated overlapping and complementing *in source* fragmentation spectra of 3PGA (4TMS) (Fig. 1). GC-EI-(TOF)MS at nominal mass resolution was abundant in low to medium molecular weight fragments (Fig. 1A). GC-APCI-(TOF)MS provided information on medium to high molecular weight fragments and molecular ions at high mass resolution (Fig. 1B). Initial saturating photosynthetic in vivo labeling experiments with *Synechocystis sp.* PCC 6803 (*Synechocystis*) provided mass spectra of 3PGA (4TMS) from maximally ^13^C-labeled *Synechocystis* cells for comparison with ambient mass spectra of cells with natural isotope abundances (NIA) of elements that were sampled before the ^13^C pulse (Fig. 1C). Labeling of 3PGA (4TMS) in constantly illuminated photobioreactors at 5% ^13^CO_2_ in synthetic air saturated at ≥60 min. Saturated ^13^C fractional enrichment (E*^13^C*) of the complete 3PGA (4TMS) molecule corrected for NIA, e.g. occurrence of 1.109% ^13^C within ambient carbon, and tracer purity, here 99% isotopically pure ^13^CO_2_, was >0.95. All E*^13^C* values reported in the following are NIA and tracer purity corrected unless stated otherwise. The photosynthetically ^13^C-labeled GC-EI-(TOF)MS mass spectra of this study matched to reference spectra of maximally ^13^C-labeled 3PGA (4TMS) that were generated independently by [U-^13^C]-glucose feeding of baker's yeast (*Saccharomyces cerevisiae*) (Birkemeyer et al. 2005) and archived by the Golm Metabolome Database (GMD, http://gmd.mpimp-golm.mpg.de/search.aspx) (Kopka et al. 2005). Mass shifts between labeled and ambient molecular ions and *in source* fragments of 3PGA (4TMS) revealed mass features, i.e. molecular, adduct, or fragment ions, that contained three or two carbon atoms (Fig. 1C; Supplementary Table S1) next to fragment ions that did not incorporate ^13^C and originated from the phosphate moiety of 3PGA and the TMS moieties both, of natural isotope compositions. TMS moieties are introduced by the chemical derivatization procedure that is required for GC analyses of 3PGA. *In source*, fragments with only one labeled carbon atom were not detectable by both technologies.

The two ionization technologies differed fundamentally in their initial molecular ionization and subsequent fragmentation reactions (Fig. 1D). GC-EI-(TOF)MS generated molecular radical ions [M]^+^. [M]^+^ readily dissociated into neutral radicals and the monitored fragment ions that retained the positive charge. Our in silico analysis of GC-EI-(TOF) mass spectra included EI-typical neutral elimination reactions subsequent to initial dissociation reactions and intramolecular rearrangements. In silico analysis of GC-APCI-(TOF)MS spectra expected abundant proton adducts [M + H]^+^. The [M + H]^+^ adduct ion was predicted to enter neutral elimination reactions (Fig. 1D). Potential isomerism, mesomerism, or charge delocalization of mass features were not considered in this study because these properties do not alter the molecular carbon-organization (Fig. 1D). The predicted molecular formula of mass features deduced from in silico analyses were validated by mass accuracy of measured monoisotopic masses and exact mass difference of neutral losses within GC-APCI-(TOF)MS *in source* fragmentation spectra. For this purpose, we used ambient and maximal in vivo ^13^C-labeled 3PGA (Supplementary Table S1). The monitored nonlabeled and labeled isotopologues typically matched to the monoisotopic masses of predicted molecular formula with an accuracy <0.0030 amu (Supplementary Table S1). The average accuracy across all predicted mass features was −0.0004 ± 0.0011 (mean ± standard deviation (SD)) (Supplementary Table S1). Observed mass differences caused by predicted adduct formations or neutral losses within the same *in source* GC-APCI-(TOF)MS fragmentation spectra matched with an accuracy <0.0012 amu and had an average of 0.0004 ± 0.0005 (mean ± SD) (Supplementary Table S1).

In silico fragmentation analysis of the observed EI and APCI induced mass spectra (Fig. 1D; Supplementary Table S1) revealed two common fragment ions at mass to charge ratios m/z = 357 and 459 amu (in the following fragments 357 and 459) that were previously observed (Kitson et al. 1996; Young et al. 2011). These fragments were generated by both GC-(TOF)MS technologies, despite the difference in the molecular ionization and subsequent fragmentation reactions.

Fragment 459 (Fig. 1D) contained all carbon atoms, i.e. 1,2,3-C_3_, of 3PGA. It was explained by CH_3_ radical dissociation from [M]^+^ (GC-EI-(TOF)MS) and by CH_4_ elimination from [M + H]^+^ (GC-APCI-(TOF)MS). These reactions were possible at multiple sites within the TMS moieties of 3PGA (4TMS). All potential reactions were predicted to be equivalent and to not alter the carbon configuration of 3PGA. These predictions were supported by a +3 amu shift after maximal ^13^C-labeling and validated by monoisotopic mass determinations within the accuracy ranges of our analyses (Fig. 1C; Supplementary Table S1). Fragment 459 had on average 13.1% base peak abundance within GC-EI-(TOF)MS spectra and 66.1% base peak abundance in GC-APCI-(TOF)MS spectra (Supplementary Table S1). Six alternative ions were predicted to contain 1,2,3-C_3_ from 3PGA. These ions were verified by a +3 amu mass shift following maximal ^13^C-labeling and accurate monoisotopic masses (Supplementary Table S1). The adduct ions among those were present only in GC-APCI-(TOF)MS. The most abundant adduct ion, [M + H]^+^ at 100% abundance, i.e. the base peak by definition, was accompanied by [M]^+^ at ∼ 0.7% base peak abundance. Presence of [M]^+^ confounded ^13^C isotopologue analysis of [M + H]^+^ because mass shifts by a ^12^C to ^13^C exchange, i.e. +1.0034 amu, were not resolved by GC-APCI-(TOF)MS from the mass shift caused by a hydrogen atom, i.e. 1.0078 amu. Alternative fragment ions including 1,2,3-C_3_ of 3PGA arose through elimination of a TMSOH-moiety, or through combinations of these reactions with the elimination of the silylated phosphate group (Supplementary Table S1). The resulting fragments were either unique to one of the GC-(TOF)MS technologies or of lower relative base peak abundance than fragment 459.

Fragment 357 resulted from C-C bond cleavage between 1-C and 2-C of 3PGA by the two different reaction modes of GC-EI-(TOF)MS and GC-APCI-(TOF)MS (Fig. 1D). Fragment 357 was predicted to originate from dissociative cleavage of a TMSCOO radical containing 1-C from [M]^+^ of 3PGA (GC-EI-(TOF)MS) or from neutral elimination of equivalent TMSCOOH from [M + H]^+^ (GC-APCI-(TOF)MS). Next to fragment 357, five alternative fragments were predicted to contain 2,3-C_2_ of 3PGA. These 2,3-C_2_ containing fragment ions originated from elimination of 1-C as CO or CO_2_ from 3PGA (4TMS) and rearrangement or from losses of 1-C combined with removal of a methyl-group or of the silylated phosphate group (Supplementary Table S1). These predictions were verified by a +2 amu shift after saturating ^13^C-labeling and confirmed by accurate monoisotopic masses (Fig. 1C; Supplementary Table S1). The five alternative 2,3-C_2_ fragment ions were either of low abundance compared to fragment 357 or absent from GC-APCI-(TOF)MS *in source* fragmentation spectra. No *in source* fragment ions containing either 1,2-C_2_ or single carbon atoms of 3PGA were discovered.

Eight fragments are reported in this study that did not receive an in vivo ^13^C-label. Five of these fragments were predicted to contain the phosphate moiety of 3PGA (4TMS); 3 fragments originated exclusively from trimethylsilyl (TMS) moieties. These fragments were used for control purposes, e.g. for quantitative analyses or were included in the mass accuracy assessments reported above (Supplementary Table S1).

### Calculation and validation of ^13^C fractional enrichment (E*^13^C*) measurements at position 1-C of 3PGA

The direct measurement of E*^13^C* at position 1-C of 3PGA by *in source* fragmentation was not possible, but positional information was available through combination of E*^13^C* from the complete 3PGA molecule and its fragmented substructures. We chose to measure E*^13^C* of 1,2,3-C_3_ (E*^13^C*_1,2,3-C3_) by fragment 459 and E*^13^C*_2,3-C2_ by fragment 357 extracting paired E*^13^C*s from the same GC-(TOF)MS files. We calculated E*^13^C*_1-C_ by Equations (1)–(3) analogous to a previous report on carbon-positional E*^13^C* analysis of aspartate (Wittemeier et al. 2024), where Equations (1) and (2) state that E*^13^C* of a structure with a known number of carbon atoms is equal to the average of E*^13^C* across all carbon positions within the structure. Equation (3) solves Equations (1) and (2) for the calculation of E*^13^C*_1-C_.


(1) $$E^{13}C_{1,2,3-C3}=(E^{13}C_{1-C}+E^{13}C_{2-C}+E^{13}C_{3-C})\times 3^{-1}$$

(2) $$E^{13}C_{2,3-C2}=(E^{13}C_{2-C}+E^{13}C_{3-C})\times 2^{-1}$$

(3) $$E^{13}C_{1-C}=3\times E^{13}C_{1,2,3-C3}\text{–}2\times E^{13}C_{2,3-C2}$$

**Figure 2.**
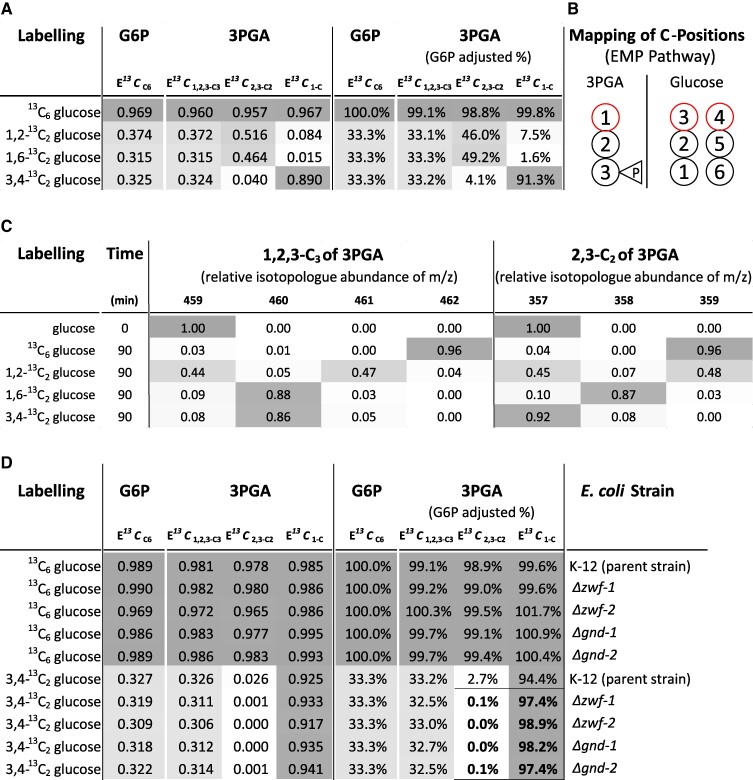
Validation of C-position specific ^13^C fractional enrichment (E*^13^C*) analyses of 3PGA by in vivo stable isotope labeling of *E. coli* with positional labeled glucoses. **A)** E*^13^C* calculations from NIA-corrected relative isotopologue distributions of intracellular G6P and 3PGA before and after adjustment, i.e. % relative to G6P, to the dilution by intracellular ^12^C and the different isotopic purities of labeled glucoses applied to *E. coli*. **B)** Mapping of carbon positions from G6P onto 3PGA according to the EMP pathway (Supplementary Fig. S1). Red outline indicates 1-C of 3PGA and its expected origin from C atoms of G6P. **C)** RIAs distributions of 1,2,3-C_3_ (3PGA_459-462) and 2,3-C_2_ (3PGA_357-359) of 3PGA after correction for NIA; data of experiment **A**). **D)** E*^13^C* position-specificity analysis of 3PGA by in vivo synthesis from 3,4-^13^C_2_ glucose using *E. coli* mutants deficient for the OPP pathway. *ΔZwf* and *Δgnd* mutants with deletions of genes coding for *GLUCOSE-6-PHOSPHATE 1-DEHYDROGENASE* and *DECARBOXYLATING 6-PHOSPHOGLUCONATE DEHYDROGENASE*, respectively, are compared to their respective parent strain. E*^13^C* calculations are as described under **(A)**. Experiments reported by **(A)** and **(C)** are performed with *E. coli* K-12 MG1655. The nonmutated knock-out parent strain of experiment **(D)** was *E. coli* K-12 BW25113. Nonlabeled, fully labeled ^13^C_6_ glucose, and positionally labeled 1,2-^13^C_2_ glucose, 1,6-^13^C_2_ glucose, or 3,4-^13^C_2_ glucose (top to bottom) were exclusive carbon sources. Intracellular G6P and 3PGA were analyzed 90 min after shift from ambient to labeled glucoses. E*^13^Cs* of 3PGA and E*^13^C_6_* of G6P were determined by GC-APCI-(TOF)MS. E*^13^C_6_* of G6P was determined by fragment 706, i.e. [M + H - CH_4_]^+^ of G6P (1MEOX) (6TMS), with molecular formula C_24_H_61_NO_9_PSi_6_^+^ and exact monoisotopic m/z 706.2694 amu. Measured E*^13^C*s (left) were adjusted (right) to theoretical complete labeling of intracellular G6P, i.e. 100.0% for ^13^C_6_ glucose feeding and to 33.3% for ^13^C_2_ glucose feedings, to account for variations of intracellular ^13^C label dilution and ^13^C tracer purity. The experiments were repeated twice independently. Data are averages across the two experiments.

We validated the position-specificity of our E*^13^C* analyses by in vivo metabolization of commercially available positional ^13^C-labeled glucoses because positional ^13^C-labeled 3PGA was not commercially available and needed to be synthesized. Positional labeled 3PGA was obtained by feeding 3,4-^13^C_2_-glucose, 1,2-^13^C_2_-glucose, 1,6-^13^C_2_-glucose and, as a control, uniformly labeled 1,2,3,4,5,6-^13^C_6_ (^13^C_6_)-glucose as exclusive carbon sources to *E. coli* strain K-12 MG1655 cultures (Fig. 2A to C). We determined and calculated E*^13^C* of 3PGA using GC-APCI-(TOF)MS and monitored ^13^C-glucose uptake into these *E. coli* cells by analyzing E*^13^C_6_* of intracellular glucose-6-phosphate (G6P). For this purpose, we selected the CH_4_ elimination reaction from [M + H]^+^ of G6P (1MEOX) (6TMS), i.e. the methoxyaminated and trimethylsilylated chemical derivative of G6P required for GC-MS metabolite profiling. The fragment ion [M + H - CH_4_]^+^ of G6P had molecular formula C_24_H_61_NO_9_PSi_6_^+^ with exact monoisotopic m/z = 706.2694 amu and was detected at expected retention time with an accuracy <0.0030 amu.

Comparing E*^13^C*_1,2,3-C3_ of 3PGA to E*^13^C*_6_ of G6P, the complete molecules of both metabolites were approximately equally labeled at 90 min after the ^13^C-glucose pulses (Fig. 2A). We corrected for in vivo variations of G6P labeling between the feeding experiments by numerically adjusting E*^13^C*_6_ of intracellular G6P to 100% when labeling with ^13^C_6_-glucose and to 33.3% when feeding ^13^C_2_-glucoses. To distinguish from the measured E*^13^C*, we report the adjusted E*^13^C* as percentages. G6P-adjusted E*^13^C*_1,2,3-C3_ of 3PGA was 99.1% after 90 min feeding of ^13^C_6_-glucose and 33.1–33.3% upon feeding ^13^C_2_-glucoses. These analyses indicated similar approximations to isotopic steady state across all feeding experiments (Fig. 2A). To interpret the biosynthesis of positional labeled 3PGA, we analyzed the carbon mapping of G6P onto 3PGA considering the relevant metabolic pathways, e.g. (Wushensky et al. 2018). We expected that in vivo metabolization of ^13^C-glucoses causes carbon-positional labeling of 3PGA that follows predominantly the carbon mapping of the EMP pathway where 3-C and 4-C of glucose are expected to constitute 1-C of 3PGA, 2-C and 5-C of glucose convert to 2-C of 3PGA, and 1-C and 6-C of glucose convert to 3-C of 3PGA (Fig. 2B; Supplementary Fig. S1). Under experimental conditions similar to our study, *E. coli* metabolized glucose with 88% ± 4% flux ratio through the EMP pathway and 11% ± 4% contribution of the OPP pathway (Hollinshead et al. 2016). The Entner–Doudoroff pathway appeared not to be used by *E. coli* and was reported to contribute a negligible flux ratio <1% (Hollinshead et al. 2016). According to expectations, 3,4-^13^C_2_-glucose labeled a single C-atom in 1,2,3-C_3_, fragment 459, of 3PGA but did not label 2,3-C_2_, fragment 357, as was evident from RIA distribution analyses (Fig. 2C). 1,6-^13^C_2_-glucose labeled one C-atom each in 1,2,3-C_3_ and in 2,3-C_2_ of 3PGA. 1,2-^13^C_2_-glucose caused incorporation of two ^13^C atoms into half of 1,2,3-C_3_ and 2,3-C_2_ from 3PGA, whereas the other half did not receive ^13^C (Fig. 2C).

The E*^13^C*_1-C_ calculations according to Equation (3) demonstrated that 91.3% (G6P-adjusted) of the ^13^C atoms from 3,4-^13^C_2_-glucose converted as expected into the 1-C position of 3PGA (Fig. 2A). Incomplete conversion of ^13^C atoms from 3,4-^13^C_2_-glucose into the 1-C position of 3PGA indicated the expected minor contribution by the OPP pathway. A similar minor contribution of the OPP pathway was evident from our feeding experiments with 1,2-^13^C_2_-glucose and 1,6-^13^C_2_-glucose. 1,2-^13^C_2_-glucose labeled 7.5% of the 1-C positions from 3PGA, 1,6-^13^C_2_-glucose labeled only 1.6% (Fig. 2A). These deviations from the carbon mapping of the EMP pathway were consistent with the alternative carbon-mapping of the OPP pathway that decarboxylates 1-C of G6P and converts 3 G6P molecules into 5 molecules of 3PGA (Supplementary Fig. S2) (Sprenger 1995; Kruger and von Schaewen 2003). 4-C, 5-C and 6-C of G6P are converted into the 1-C, 2-C, and 3-C positions of three among the five 3PGA molecules. Consequently, the 6-^13^C atom of G6P was not expected to label 1-C of 3PGA. The two other 3PGA molecules generated by the OPP pathway originate from rearrangement of 2-C and 3-C from G6P through transaldolase and transketolase reactions. These two 3PGA molecules have either 2-C or 3-C of G6P at 1-C position (Supplementary Fig. S2). Consequently, the higher E*^13^C*_1-C_ of 3PGA after 1,2-^13^C_2_-glucose feeding compared to 1,6-^13^C_2_-glucose and the incomplete conversion of 3,4-^13^C_2_-glucose into 1-C of 3PGA were explained by the carbon mapping of the OPP pathway.

In order to verify this conclusion and to prove position-specificity of our E*^13^C* analyses, we analyzed *Δzwf* and *Δgnd* mutants of *E. coli* that have an inactive OPP pathway. The *GLUCOSE-6-PHOSPHATE 1-DEHYDROGENASE* (*ZWF*) and *DECARBOXYLATING 6-PHOSPHOGLUCONATE DEHYDROGENASE* (*GND*) genes code for the enzymes ZWF (EC: 1.1.1.49) and GND (EC: 1.1.1.44), both essential for the OPP pathway. Two independent knock-out mutant strains of each gene were compared to their respective K-12 parent strain BW25113, feeding 3,4-^13^C_2_-glucose or ^13^C_6_-glucose (Fig. 2D). The ^13^C_6_-glucose experiments demonstrated isotopic steady-state of G6P and 3PGA across all mutants and between the alternative *E. coli* K-12 strains (Fig. 2A and D) and complete labeling of all positions. Our conclusion was supported and position-specificity of E*^13^C*_1-C_ and E*^13^C*_2,3-C2_ analyses of 3PGA demonstrated in the absence of the OPP pathway by feeding 3,4-^13^C_2_-glucose (Fig. 2D). Resulting E*^13^C*_2,3-C2_ of the *Δzwf* and *Δgnd* mutants averaged at 0.04% and E*^13^C*_1-C_ at 97.96%. The parent strain BW25113 had E*^13^C*_2,3-C2_ 2.67% and E*^13^C*_1-C_ 94.38% in comparison. C-remodeling through the OPP pathway without mutations was similar as observed by *E. coli* strain K-12 MG1655 (Fig. 2A).

### E*^13^C*_1-C_ analyses of 3PGA reflect the canonical ^13^CO_2_ assimilation mechanism of RUBISCO

**Figure 3.**
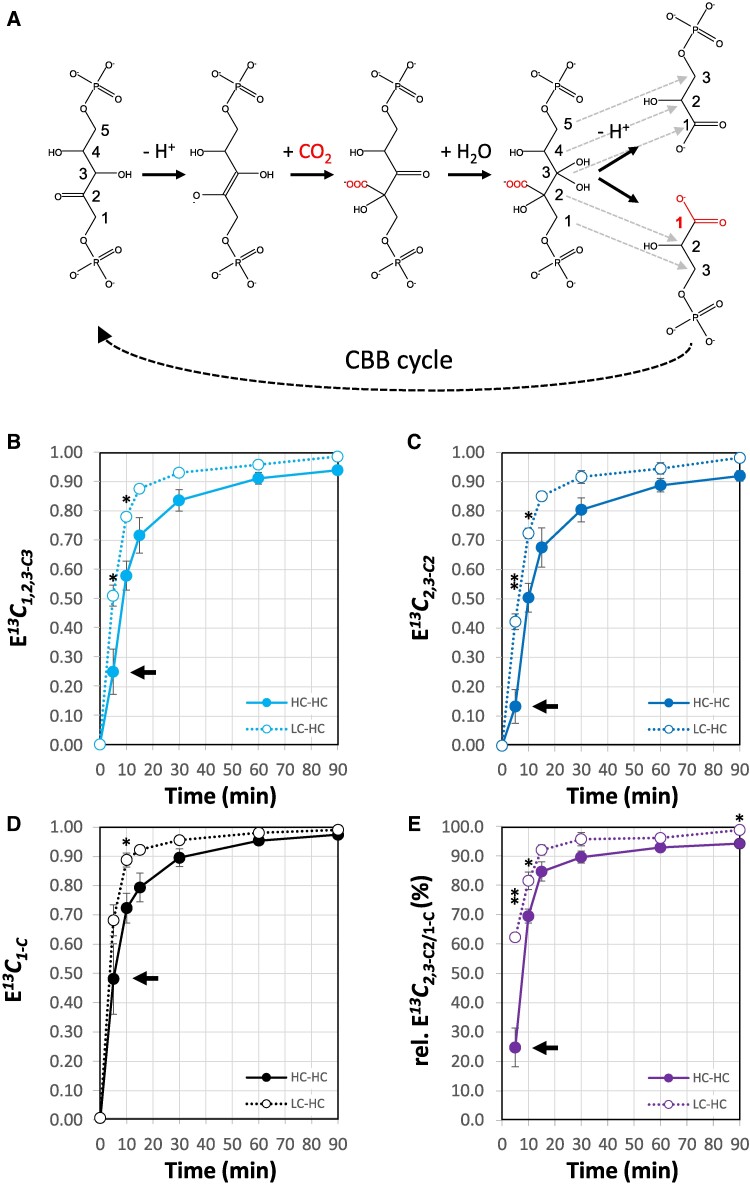
Positional ^13^C fractional enrichment (E*^13^C*) analysis of 3PGA from dynamic ^13^CO_2_ labeling experiments of *Synechocystis sp.* PCC 6803. **A)** Elementary reaction steps of the carboxylation mechanism of RUBISCO with carbon position mapping between ribulose-1,5-bisphosphate (RuBP), CO_2_ and 2 molecules of 3PGA (dashed arrows). The assimilated carbon atom (red) is incorporated into the 1-C position of one 3PGA molecule and rearranged through the CBB cycle. **B)** E*^13^C*_1,2,3-C3_ of 3PGA (4TMS) fragment 459. **C)** E*^13^C*_2,3-C2_ of 3PGA (4TMS) fragment 357. **D)** E*^13^C*_1-C_ of 3PGA calculated by Equation (3). **E)** Relative E*^13^C*_2.3-C2/1-C_ (%) calculated by Equation (4). *Synechocystis* cells were preacclimated to low CO_2_ of ambient air (LC, ∼0.04%) or to high CO_2_ (HC, 5.0%). Preacclimated cells were probed by a 5.0% ^13^CO_2_ pulse to generate ^13^C-labeling time courses, nonsteady-state LC-HC (open circles), or steady-state, HC-HC (closed circles), over a period of 0 to 90 min after pulse initiation. Three independent experiments of HC- and LC-preacclimated cells were performed in photobioreactors and analyzed by GC-APCI-MS (means ± SE). Significant differences between HC and LC cells are indicated by asterisks, * *P* ≤ 0.05, or ** *P* ≤ 0.01 (Student’s *t*-test). Inserted arrows highlight differences of E*^13^C* in the complete 3PGA molecule, E*^13^C*_1,2,3-C3_, compared to E*^13^C*_2,3-C2_ and E*^13^C*_1-C_  **(B and C)**. Relative E*^13^C*_2.3-C2/1-C_ (%) **(E)** demonstrates nonhomogenous labeling of 3PGA at early time points consistent with the RUBISCO reaction mechanism **(A)** and subsequent redistribution of assimilated ^13^C into carbon positions 2,3-C_2_. Data are means ± SE, *n* = 3 biological replicates.

Positional E*^13^C*_1-C_ analysis of 3PGA enables in vivo measurements of CO_2_ assimilation through RUBISCO. The carboxylation reaction mechanism of RUBISCO generates two molecules of 3PGA from RuBP and incorporates CO_2_ into 1-C position of one of these 3PGA molecules (Fig. 3A) (Douglas-Gallardo et al. 2022; Prywes et al. 2023). This 3PGA molecule contains 1,2-C_2_ of RuBP that map inversely to 2,3-C_2_ of 3PGA. The second molecule of 3PGA contains 3,4,5-C_3_ of RuBP that map to 1,2,3-C_3_ of 3PGA. RuBP is regenerated from 3PGA through the CBB cycle. In *Synechocystis* similar to plants, gluconeogenetic, transaldolase, and transketolase reactions (Makowka et al. 2020), rearrange the carbon configuration and redistribute part of the initial assimilated carbon atoms to positions within RuBP that generate 2,3-C_2_ of 3PGA. As CBB reactions follow upon the initial RUBISCO reaction, we expected for the chosen non-RUBISCO-limiting high 5.0% CO_2_ pulse, a time lag that causes nonhomogenous 3PGA labeling during the initial phase of dynamic photosynthetic ^13^CO_2_ labeling, where 1-C of 3PGA labels more rapidly than 2,3-C_2_. Differential labeling kinetics within the carbon backbone of 3PGA can be characterized by the ratio of E*^13^C*_2,3-C2_ relative to E*^13^C*_1-C_. In this study, we defined this relative fractional enrichment as percentage according to Equation (4).


(4) $$\mathrm{rel}.\;E^{13}C_{2,3-C2/1-C}(\text{\%})=E^{13}\;C_{2,3-C2}\times E^{13}C_{1-C}^{-1}\times 100$$

At the start of a dynamic photosynthetic ^13^CO_2_ pulse, relative E*^13^C*_2.3-C2/1-C_ (%) was expected to approximate 0%. Subsequently relative E*^13^C*_2.3-C2/1-C_ (%) will approximate 100% as the 3PGA pool approaches maximal ^13^C labeling through the CBB cycle reactions that follow upon the initial RUBISCO catalyzed carbon assimilation. Mobilization of prior-to-pulse accumulated, ambient carbohydrates for anaplerotic RuBP regeneration (Makowka et al. 2020) will cause intermediate isotopic steady-states at relative E*^13^C*_2,3-C2/1-C_ (%) < 100% depending on the rate of the anaplerotic reactions. To test these expectations by our methodology, we designed experiments in constantly illuminated photobioreactors to preacclimate *Synechocystis* either to LC (active CCM and low internal organic carbon storage) or to HC (mostly inactive CCM and high internal organic carbon storage), and probed both cultivations by identical dynamic ^13^CO_2_ labeling at 5.0% (v) HC. We kept pulse labeling conditions of the differentially acclimated cells identical to avoid differences of CO_2_ or HCO_3_^−^ diffusion and accumulation within the liquid growth medium. In the following, the steady-state HC-HC experiments were compared to the nonsteady-state LC-HC shift condition expecting a differentially active CCM within the first hour after the pulse.

*Synechocystis* cells rapidly incorporated ^13^C into the complete 3PGA molecule when exposed to a HC (^13^CO_2_) pulse in a photobioreactor (Fig. 3B). Irrespective of preacclimation, E*^13^C*_2,3-C2_ was consistently smaller than E*^13^C*_1,2,3-C3_ and consequently E*^13^C*_1-C_ larger, especially during the initial pulse phase at <30 min, confirming expected nonhomogenous in vivo labeling of 3PGA (Fig. 3B to D). Homogenous labeling of 3PGA was approximated but remained incomplete even at ≥60 min after the pulse; E*^13^C*_1-C_ exceeded E*^13^C*_2,3-C2_ even at fractional enrichments >0.90 (Fig. 3C and D). In agreement with these observations, relative E*^13^C*_2,3-C2/1-C_ (%) was initially low and approximated saturation at >90% across the course of the labeling pulse (Fig. 3E). Comparing the LC-HC shift experiments to the HC-HC control, we detected significant differences of E*^13^C* caused by preacclimation at ≤10 min. 3PGA from LC-acclimated cells labeled more rapidly and had consistently higher E*^13^C*_1-C_, E*^13^C*_2.3-C2_, and E*^13^C*_1,2,3-C3_ (Fig. 3B to D). Similarly, relative E*^13^C*_2.3-C2/1-C_ (%) of the LC-HC shift experiment was higher than the HC-HC control throughout the monitored period of pulse labeling. Differences of relative E*^13^C*_2.3-C2/1-C_ (%) tested significant (*P* ≤ 0.05, Student’s *t*-test) at 5, 10, and 90 min (Fig. 3E).

### Quantification of molar ^13^C assimilation into 1-C of 3PGA by combined GC-EI-(TOF)MS and GC-APCI-(TOF)MS analyses

Next to changes of reactions rates, E*^13^C* kinetics depends on changes of cellular metabolite concentrations. Such changes can be expected for 3PGA upon acclimation to different Ci supply and may occur in mutants or during nonsteady-state conditions, such as the LC-HC shift of this study. To account for metabolite concentration changes, we determined next to E*^13^C*, the molar concentrations of 3PGA (C_3PGA_) of each sample. We measured all samples by GC-EI-(TOF)MS and GC-APCI-(TOF)MS and chose the optimal technology to obtain the two required parameters. Multiplication of E*^13^C* by the metabolite concentration calculates the molar concentration of ^13^C (C*^13^C*) incorporated into the complete molecule (C*^13^C*_1,2,3-C3_) or into specified carbon positions (C*^13^C*_2,3-C2_, or C*^13^C*_1-C_).

**Figure 4.**
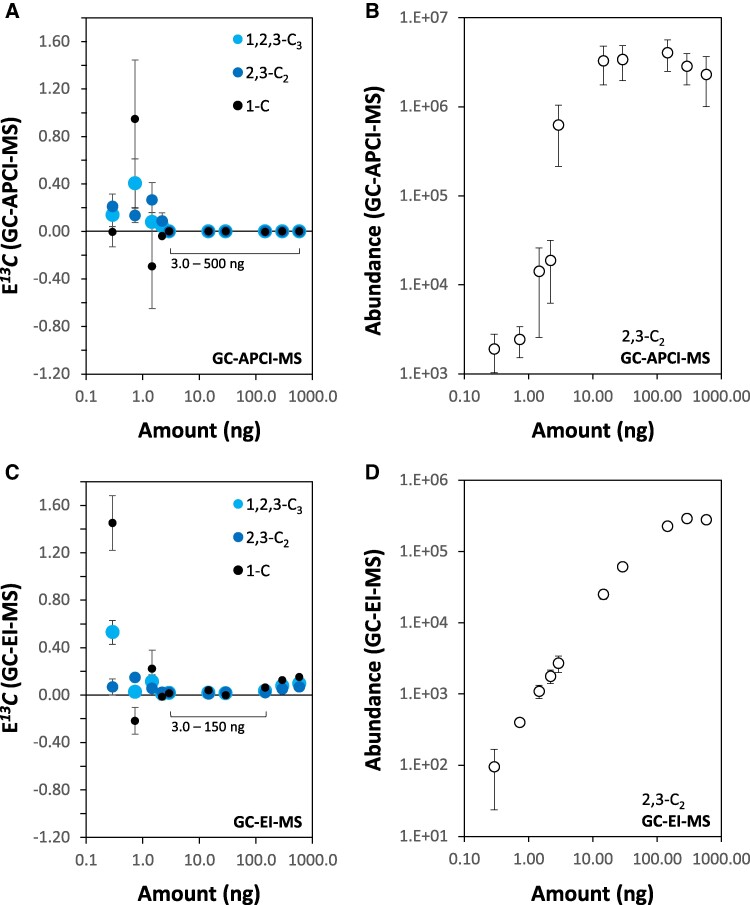
Concentration range for accurate NIA-correction of calculated ^13^C fractional enrichment, E*^13^C*_1-C_, of 3PGA and of measured E*^13^C*_1,2,3-C3_ and E*^13^C*_2,3-C2_ from paired GC-EI-(TOF)MS and GC-APCI-(TOF)MS analyses. **A)** NIA-corrected E*^13^C*_1-C_, E*^13^C*_2,3-C2_, and E*^13^C*_1,2,3-C3_ of 3PGA (4TMS) determined through isotopologue distributions of fragment 357 and fragment 459 measured by GC-APCI-(TOF)MS. **B)** Quantitative calibration of 3PGA abundance using NIA-corrected isotopologue distributions of 3PGA (4TMS) fragment 357 measured by GC-APCI-(TOF)MS. **C)** NIA-corrected E*^13^C*_1-C_, E*^13^C*_2,3-C2_, and E*^13^C*_1,2,3-C3_ of 3PGA (4TMS) determined through isotopologue distributions of fragment 357 and fragment 459 measured by GC-EI-(TOF)MS. **D)** Quantitative calibration of 3PGA abundance using NIA-corrected isotopologue distributions of 3PGA (4TMS) fragment 357 measured by GC-EI-(TOF)MS. Quantitative calibration series of 3PGA were prepared in independent triplicates from ambient, nonlabeled, chemically pure 3PGA reference substance. Paired analyses of the same samples were performed by GC-EI-(TOF)MS and GC-APCI-(TOF)MS. The amount of 3PGA in 1 µL sample injected into the GC-(TOF)MS systems in splitless mode was plotted against respective arbitrary abundance units. Note that accurate NIA-correction is limited at low 3PGA concentrations by increasing contributions of noise to isotopologue distribution measurements. At the upper limit of abundance quantification saturation of the most abundant isotopologues may affect accuracy of NIA-correction. Ranges of accurate NIA-correction are reported by inserts **(A)** and **(C)**. Visualized data are means ± SE, *n* = 3 replicates.

E*^13^C* was determined, as in all analyses reported above, by GC-APCI-(TOF)MS considering first, the robust NIA-correction across a large range of 3PGA abundances (Fig. 4A). As expected, E*^13^C*_1,2,3-C3_, E*^13^C*_2,3-C2_, and E*^13^C_1-C_* of nonlabeled, pure 3PGA reference compound approximated E*^13^C* = 0 within the linear ranges of abundance measurements by both GC-(TOF)MS technologies (Fig. 4B and D). GC-EI-(TOF)MS measurements were valid between 3.0 and 150.0 ng 3PGA injected (Fig. 4C), while GC-APCI-(TOF)MS extended the range of accurate natural E*^13^C* determinations into abundance saturation and was valid at 3.0 to 500.0 ng 3PGA injected (Fig. 4A). Beyond the low and high abundance limits, E*^13^C* was overestimated by both technologies (Fig. 4A and C) and highly variable at low 3PGA concentrations.

Other than pure reference compounds, complex biological samples may contain metabolites that interfere with E*^13^C* determination through coelution and partially or completely overlapping isotopologue distributions. To test for such interference, we analyzed E*^13^C* of nonlabeled, endogenous 3PGA from the complex primary metabolome of the cyanobacteria *Synechocystis* and *Microcystis aeruginosa* PCC 7806. *M. aeruginosa* PCC 7806 was chosen arbitrarily as an alternative biological material, so as to highlight the necessity of a careful case by case validation of biological matrices. Within the assessed abundance limits of the two GC-(TOF)MS technologies (Fig. 4), natural E*^13^C* of 3PGA from these complex samples approximated but were not exactly equal to zero (Supplementary Table S2). Determination by high-mass resolution GC-APCI(TOF)MS was more accurate at E*^13^C* < 0.001 than measurements by nominal-mass-resolution GC-EI(TOF)MS that overestimated at E*^13^C* < 0.03. Differences between the technologies became more apparent using complex metabolite samples from cyanobacteria. Natural E*^13^C* of 3PGA measured by GC-APCI(TOF)MS remained accurate approximations to zero. In contrast, GC-EI(TOF)MS measurements overestimated to a similar degree as pure 3PGA in the case of *M. aeruginosa* or revealed additional interference in the case of *Synechocystis* (Supplementary Table S2). Interferences that only arise in vivo through label-induced mass shifts are difficult to assess within complex biological samples and require careful case by case manual supervision.

**Figure 5.**
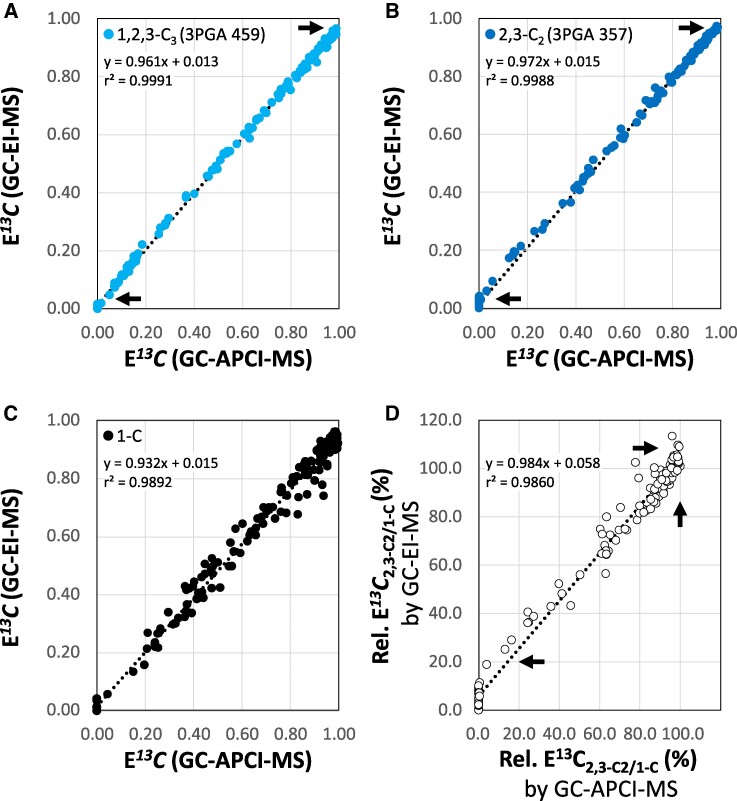
Correlation of NIA-corrected ^13^C fractional enrichment (E*^13^C*) measurements of 3PGA from ^13^CO_2_ dynamic pulse labeling experiments with *Synechocystis sp.* PCC 6803. GC-EI-(TOF)MS results are compared to paired GC-APCI-(TOF)MS measurements of the same chemically derivatized samples. **A)** Fragment ion 459 monitoring E*^13^C*_1,2,3-C3_ (light blue) of 3PGA. **B)** Fragment ion 357 monitoring E*^13^C*_2,3-C2_ (dark blue) of 3PGA. **C)** E*^13^C*_1-C_ of 3PGA calculated by Equation (3). **D)** Relative E*^13^C*_2,3-C2/1-C_ (%) calculated by Equation (4). The analysis combines data of 24 dynamic labeling experiments (166 samples) from 0 min (immediately before) to 90 min after the ^13^C-pulse. GC-EI-(TOF)MS compared to GC-APCI-(TOF)MS overestimated E*^13^C* of low-labeled samples **(A** and **B**; arrows at panel bottom) and underestimated E*^13^C* of highly labeled samples **(A** and **B**; arrows at panel top). Relative E^13^C_2,3-C2/1-C_ (%) was overestimated by GC-EI-(TOF)MS relative to GC-APCI-(TOF)MS **(D**; bottom and top arrows). GC-APCI-(TOF)MS measurements of relative E^13^C_2,3-C2/1-C_ (%) did not exceed 100% **(D**; vertical arrow). Inserts report linear regression functions and r² of Pearson’s correlation coefficients.

We expected differences of E*^13^C* measurements between low (GC-EI-(TOF)MS) and high (GC-APCI-(TOF)MS) mass resolution mass spectrometry as the later avoids interferences of equal nominal but different exact mass. To assess such differences, we correlated E*^13^C* determined by GC-APCI-(TOF)MS to paired GC-EI-(TOF)MS measurements of >100 differentially labeled, complex samples from *Synechocystis* that ranged from zero to maximum E*^13^C* of 3PGA (Fig. 5). E*^13^C* measurements by GC-APCI-(TOF)MS and GC-EI-(TOF)MS were highly correlated with linear Pearson’s correlation coefficients r² > 0.998 for both fragment 357 and fragment 459, but the slopes of linear regression functions did not equal 1.0 and intercepts had an offset relative to the origin (Fig. 5A and B). The two technologies deviated at low and high E*^13^C*, especially in regard to the calculated parameters, E*^13^C*_1-C_ and E*^13^C*_2.3-C2/1-C_ (%). As expected from the previous analyses (Fig. 4; Supplementary Table S2), GC-EI-(TOF)MS overestimated E*^13^C* of the fragments 357 and 459 at low E*^13^C* (Fig. 5A to C) and GC-EI-(TOF)MS reported higher E*^13^C*_2.3-C2/1-C_ (%) than GC-APCI-(TOF)MS (Fig. 5D). At maximum E*^13^C*, GC-EI-(TOF)MS underestimated relative to GC-APCI-(TOF)MS (Fig. 5A to C). Unexpectedly, E*^13^C*_2.3-C2/1-C_ (%) calculations from GC-EI-(TOF)MS exceeded 100% at maximum ^13^C-labeling. E*^13^C*_2.3-C2/1-C_ (%) cannot exceed 100% during photosynthetic pulse labeling experiments because 1-C of 3PGA assimilates ^13^C first. In agreement with this expectation, E*^13^C*_2.3-C2/1-C_ (%) determined by GC-APCI-(TOF)MS never exceeded the expected 100% limit (Fig. 5D).

We assessed accuracy and precision of E*^13^C* measurements by GC-APCI-(TOF)MS using, nonlabeled glucose with expected and measured E*^13^C* = 0.000 (Supplementary Table S3), single position labeled ^13^C_1_-glucoses, and fully labeled ^13^C_6_-glucose analyzed as pure reference substance. In addition, we analyzed fully labeled ^13^C_6_-sorbitol that was added as internal standard to our primary metabolome preparations from *Synechocystis* (Supplementary Table S3). We used reference compounds with >99% isotopic purity and measured E*^13^C* of fragment ions containing all 6 carbon atoms [M + H - CH_4_]^+^ or 3 and 4 carbon atoms, respectively (Supplementary Table S3). E*^13^C* measurements by GC-APCI-(TOF)MS were accurate within the limits of the manufacturer’s analysis certificates, e.g. E*^13^C* = 0.9945 of [M + H - CH_4_]^+^ from ^13^C_6_-glucose, E*^13^C* = 0.1667 from ^13^C_1_-glucoses, and E*^13^C* = 0.9994 from ^13^C_6_-sorbitol (Supplementary Table S3). The precision of E*^13^C* was <0.001 SD from pure reference compounds or <0.005 SD of ^13^C_6_-sorbitol from the complex samples (Supplementary Table S3).

C_3PGA_ was determined by GC-EI-(TOF)MS. This technology extended the linear range of abundance quantitation compared to GC-APCI-(TOF)MS (Fig. 4). The NIA-corrected sum of isotopologues from fragment 357 measured by GC-EI-(TOF)MS provided a linear range of quantification between 1 and 150 ng 3PGA injected (Fig. 4D). The GC-APCI-(TOF)MS abundance of this fragment was saturated beyond ∼10 ng (Fig. 4B). We chose fragment 357 for this comparison because it had similar relative base peak abundances when monitored by the two GC-(TOF)MS technologies, namely 37.3% by GC-EI-(TOF)MS and 25.6% by GC-APCI-(TOF)MS (Supplementary Table S1). GC-EI-(TOF)MS based abundance measurements using the NIA-corrected sum of isotopologues from fragment 357 were highly matched to the respective sum of isotopologues from fragment 459 or to the fragment ions 299 and 315 that did not receive ^13^C label. Relative abundances of these four fragments correlated with Pearson’s correlation coefficients r > 0.996 across complex samples (*n* = 168) from dynamic ^13^CO_2_ labeling experiments of *Synechocystis* cells (Supplementary Table S4). Quantifications of 3PGA by each of the fragment ions had similar relative standard deviations (RSD) from the means of biological replicate groups within our 168 analyses (Supplementary Table S4). RSDs of nmol (3PGA) * OD_750_^−1^ * mL^−1^ quantified by fragment 357 ranged from 9.5% to 13.2% across steady-state (HC-HC) conditions and from 11.3% to 20.6% indicative of an expected larger variation during a LC-HC state transition (Supplementary Table S4). Because fragment 459 was less abundant and had slightly increased RSDs (Supplementary Table S4) compared to fragment 357, all subsequent quantifications of 3PGA concentrations were through the NIA-corrected sum of isotopologue abundances from fragment 357. Fragments 299 and 315 provided in part improved RSDs but were used in the following only as mass spectral qualifiers for correlation checks but not for abundance quantification. This decision was made, because fragments 299 and 315 are common to all phosphorylated compounds present in our complex samples and thereby less specific.

### Molar CO_2_ assimilation rates into 1-C and 2,3-C_2_ of 3PGA demonstrate differential metabolic functions of the divergent GAPDHs from *Synechocystis*

**Figure 6.**
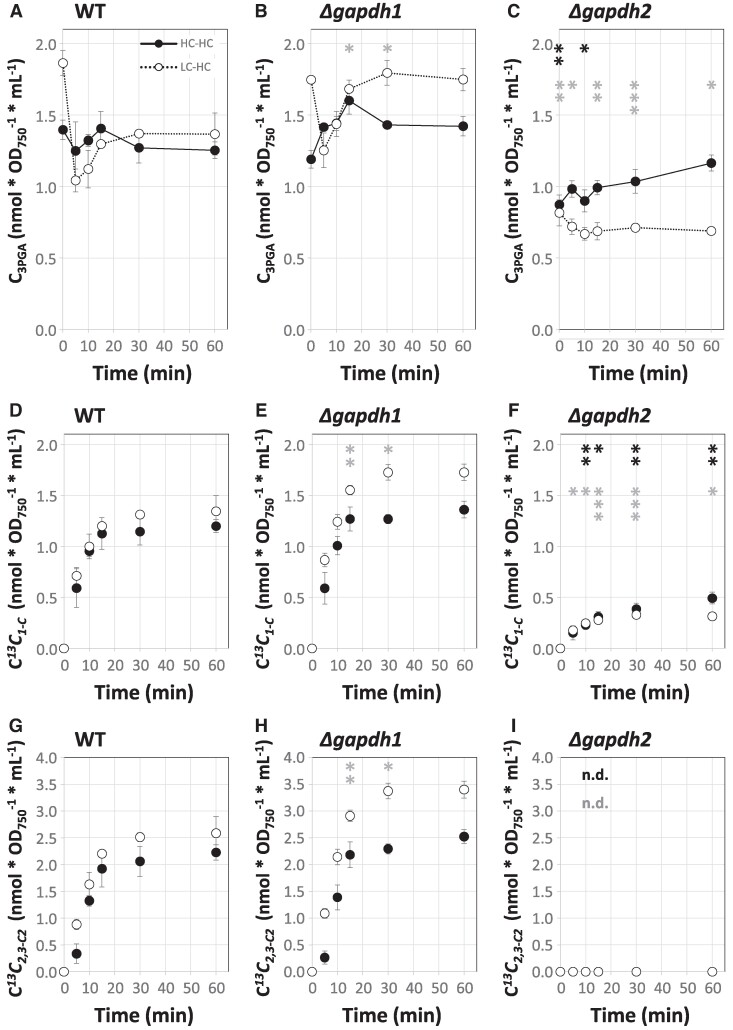
Carbon assimilation into positions 1-C and 2,3-C_2_ of 3PGA of high CO_2_ (HC, 5.0%) and low CO_2_ (LC, ambient) preacclimated WT *Synechocystis sp.* PCC 6803 compared to *Δgapdh1* and *Δgapdh2* mutant cells. **A)–C)** 3PGA concentrations, C_3PGA_ (nmol * OD_750_^−1^ * mL^−1^) quantified by GC-EI-MS technology. **D)–F)** Positional carbon assimilation of molar ^13^C concentrations, C*^13^C_1-C_*, in units of nmol (^13^C) * OD_750_^−1^ * mL^−1^. **G)–I)** Positional carbon assimilation of C*^13^C_2,3-C2_*. Cells were probed by a 5.0% ^13^CO_2_ (HC) pulse to generate either LC-HC (open circles, dashed lines, nonsteady-state) or HC-HC (closed circles, solid lines, steady-state) dynamic labeling time series. Positional carbon assimilation of each sample was calculated from C_3PGA_ and E*^13^C* data of paired GC-EI-(TOF)MS and GC-APCI-(TOF)MS analyses (Supplementary Table S5). Three independent experiments of HC- and LC-preacclimated cultures were performed in photobioreactors. Data are means ± SE, *n* = 3 biological replicates. Significant differences between mutant and WT cells are indicated by black (HC-HC) or gray (LC-HC) asterisks, * *P* ≤ 0.05, ** *P* ≤ 0.01, and *** *P* ≤ 0.001 (heteroscedastic, two-tailed Student’s *t*-test). Note, *Δgapdh2* mutant cells are not viable under photoautotrophic conditions and had to be precultivated in the presence of 10 mM nonlabeled glucose added to BG11 medium. The ^13^CO_2_ (HC) pulse was in all cases, in the absence of external glucose. C*^13^C_2,3-C2_* was not detectable (n.d.) in *Δgapdh2* cells.

With a method in place that quantified molar concentrations of 3PGA (C_3PGA_) and positional E*^13^C* from each sample, we calculated the molar ^13^C concentrations, C*^13^C*_1,2,3-C3_, C*^13^C*_2,3-C2_, and C*^13^C*_1-C_, of *Synechocystis* WT and of the gene deletion mutants, *Δgapdh1* and *Δgapdh2*, of the two GAPDHs from *Synechocystis* (Koksharova et al. 1998; Lucius et al. 2022; Schulze et al. 2022). With all three genotypes we performed steady-state HC-HC and nonsteady-state LC-HC shift experiments in continuous light (Fig. 6; Supplementary Table S5). The *Δgapdh2* mutant was viable only under mixotrophic conditions and other than photoautotrophic WT and *Δgapdh1* mutant, had to be precultivated in the presence of glucose (Schulze et al. 2022). To maintain comparability, all three genotypes were ^13^CO_2_ pulse labeled after liquid media exchange in the absence of glucose.

Under HC-HC conditions, C_3PGA_ did not significantly differ between WT and *Δgapdh1*. WT had 1.32 ± 0.04 (standard error (SE), *n* = 18) nmol * OD_750_^−1^ * mL^−1^ and *Δgapdh1* 1.42 ± 0.04 (SE, *n* = 18) nmol * OD_750_^−1^ * mL^−1^, respectively, averaged across 0–60 min of the HC-HC experiment. C_3PGA_ of *Δgapdh2* was significantly lower than WT with 0.99 ± 0.03 (SE, *n* = 18) nmol * OD_750_^−1^ * mL^−1^ (Fig. 6A to C; Supplementary Table S5). During the LC-HC shift C_3PGA_ of WT readjusted within the first 5 min to HC levels (Fig. 6A). A ∼1.3-fold increased C_3PGA_ after LC-preacclimation relative to HC preacclimation was expected (Orf et al. 2015). Unlike WT, *Δgapdh1* did not readjust the LC-preacclimated C_3PGA_ to HC levels within the monitored 60 min after shift and remained significantly increased (Fig. 6B). C_3PGA_ of LC-preacclimated *Δgapdh2* did not differ from its HC state and remained significantly lower than WT after LC-HC shift (Fig. 6C).

**Figure 7.**
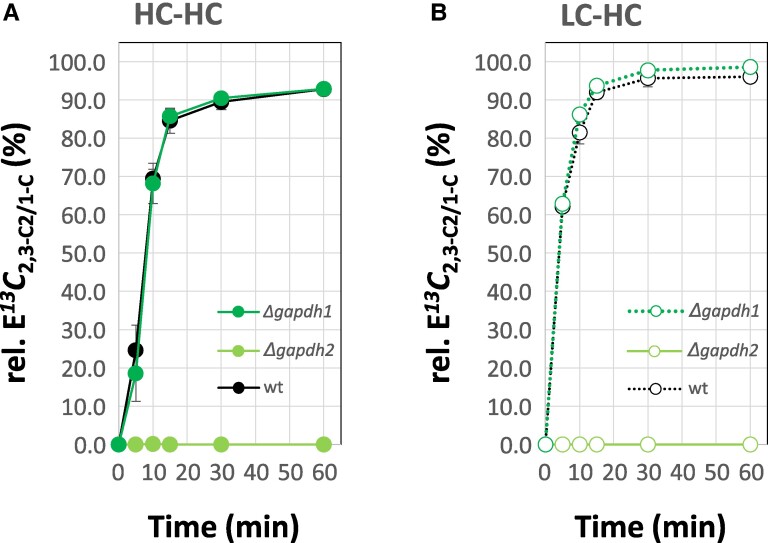
Differential labeling kinetics within the carbon backbone of 3PGA characterized by the ratio of ^13^C fractional enrichment, E*^13^C*_2,3-C2_, relative to E*^13^C*_1-C_, i.e. relative E*^13^C*_2,3-C2/1-C_ (%).WT *Synechocystis sp.* PCC 6803 (black) cells were compared to *Δgapdh1* (dark green) and *Δgapdh2* (light green) mutant cells. **A)** Relative E*^13^C*_2,3-C2/1-C_ (%) of cells preacclimated to high CO_2_ (HC, 5.0%). **B)** Relative E*^13^C*_2,3-C2/1-C_ (%) of cells preacclimated to low CO_2_ (LC, ambient). The data of Fig. 6 were used to calculate relative E*^13^C*_2,3-C2/1-C_ (%). For experimental details refer to legends of Fig. 6 and Supplementary Table S5. E*^13^C_2,3-C2_* was not detectable (n.d.) in *Δgapdh2* cells. Data are means ± SE, *n* = 3 biological replicates.

The ^13^C assimilation into 1-C of 3PGA (C*^13^C*_1-C_) at HC-HC steady-state did not significantly differ between *Δgapdh1* and WT (Fig. 6D and E). Upon LC-HC shift WT C*^13^C*_1-C_ kinetics were unchanged compared to the HC-HC steady-state but *Δgapdh1* assimilated more C*^13^C*_1-C_ (Fig. 6E). C*^13^C*_2,3-C2_ assimilation of WT and *Δgapdh1* was in both cases highly similar to C*^13^C*_1-C_ (Fig. 6G and H). Analysis of E*^13^C*_2.3-C2/1-C_ (%) as an indicator of relative changes between RUBISCO and CBB cycle activity demonstrated an almost exact match between *Δgapdh1* and WT (Fig. 7A and B). The *Δgapdh2* mutant, surprisingly, assimilated ^13^C into 1-C of 3PGA (Fig. 6F). C*^13^C*_1-C_ of *Δgapdh2* did not vary between preacclimation conditions and was ∼0.27-fold compared to WT, averaged across both conditions and the complete duration of the ^13^C pulse (Supplementary Table S5). ^13^C remained delimited in *Δgapdh2* to the 1-C position of 3PGA; C*^13^C*_2,3-C2_ of the *Δgapdh2* mutant was not detectable (Figs. 6I and 7).

**Figure 8.**
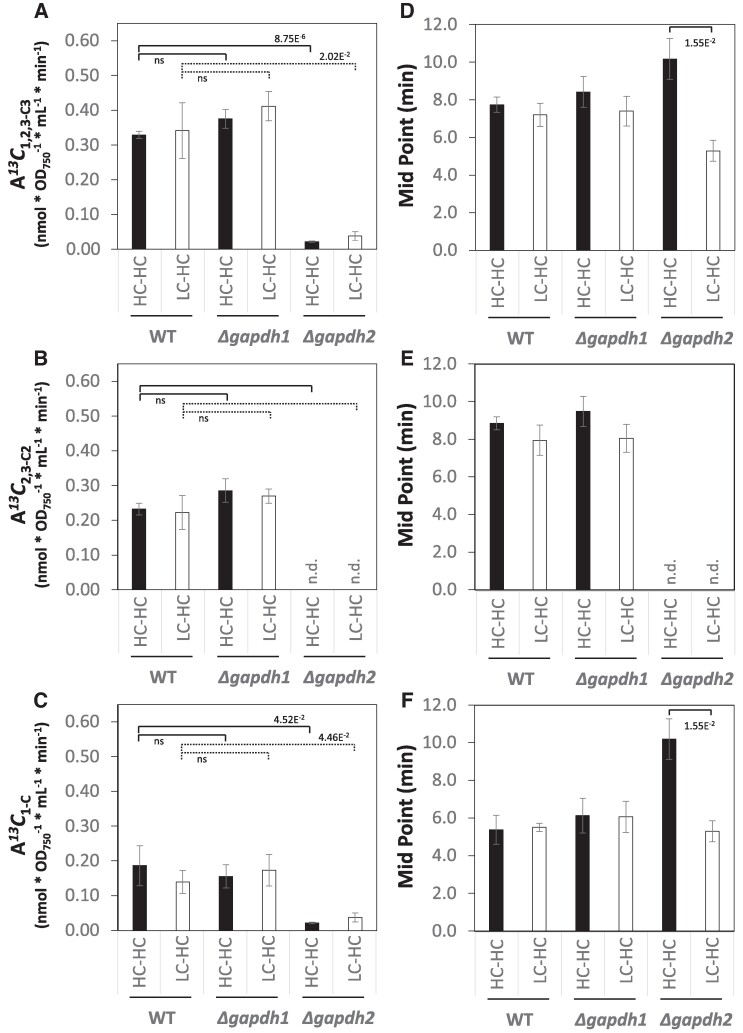
Assimilation rates (A*^13^C*) into positions 1,2,3-C_3_, 2,3-C_2_, and 1-C of 3PGA of high CO_2_ (HC, 5.0%) and low CO_2_ (LC, ambient) preacclimated WT *Synechocystis sp.* PCC 6803 compared to *Δgapdh1* and *Δgapdh2* mutant cells. **A)** A*^13^C*_1,2,3-C3_ (nmol * OD_750_^−1^ * mL^−1^ * min^−1^) of 3PGA. **B)** A*^13^C*_2,3-C2_ (nmol * OD_750_^−1^ * mL^−1^ * min^−1^) of 3PGA. **C)** A*^13^C*_1-C_ (nmol * OD_750_^−1^ * mL^−1^ * min^−1^) of 3PGA. **D)–F)** Midpoint times (min) of the logistic functions of **A)–C**), respectively. Cells were probed by a 5.0% ^13^CO_2_ (HC) pulse to generate either LC-HC, nonsteady-state, or HC-HC, steady-state, dynamic labeling time series. Assimilation rates were obtained from midpoint slopes of fitted logistic sigmoidal functions, brackets indicate Student’s *t*-test results, ns (nonsignificant), *P* < 0.05 (Supplementary Table S6); n.d., not detected. Data are means ± SE, *n* = 3 biological replicates.

The kinetic molar ^13^C assimilation measurements (Fig. 6D to H) were fitted with high significance to assumptions of logistic sigmoidal functions (Supplementary Table S6). We used the slopes at midpoint of fitted logistic sigmoidal functions to estimate average molar ^13^C assimilation rates (A*^13^C*) from the single time course measurements of our replicate cultures (Fig. 8). Alternatively, we estimated A*^13^C* as initial slopes at t_0_ of exponential regression functions (Supplementary Table S6). Both estimates were approximately equivalent in magnitude and yielded robust significance-test results (Supplementary Fig. S3). Manual inspection of initial E*^13^C* kinetics from our HC-HC experiments indicated a lag-phase of ^13^C incorporation that is not accurately modeled by exponential regression (Fig. 3B and C). These observations indicated that diffusion of ^13^Ci on its path to RUBISCO and additional diffusion due to subsequent metabolization of assimilated ^13^C, e.g. the reconfiguration of the initially assimilated 1-^13^C into the 2,3-C positions of 3PGA by the CBB cycle, must be considered. These considerations caused us to prefer logistic regression for our subsequent analyses. The rate of molar ^13^C assimilation into the complete 3PGA molecule (A*^13^C*_1,2,3-C3_) of all analyzed genotypes did not significantly differ between HC-HC steady-state and LC-HC shift conditions (Fig. 8A). A*^13^C*_1,2,3-C3_ of the WT were 0.33 ± 0.01 and 0.34 ± 0.08 (SE; *n* = 3) nmol * OD_750_^−1^ * mL^−1^ min^−1^, after HC- or LC-preacclimation, respectively. A*^13^C*_1,2,3-C3_ of *Δgapdh1* did not significantly differ from WT (Fig. 8A; Supplementary Table S6). The *Δgapdh2* mutant, in contrast, assimilated at a significantly lower rate than WT with rates of A*^13^C*_1,2,3-C3_ equal to 0.02 ± 0.002 and 0.04 ± 0.01 (SE; *n* = 3) nmol * OD_750_^−1^ * mL^−1^ min^−1^ of the HC-HC and LC-HC conditions, respectively.

The positional assimilation rates A*^13^C*_1-C_ and A*^13^C*_2,3-C2_ of WT and *Δgapdh1* did again not differ and were not significantly affected by HC- or LC-preacclimation (Fig. 8B and C; Supplementary Table S6). WT assimilated on average across both preacclimation conditions with A*^13^C*_1-C_ rate equal to 0.16 ± 0.03 (SE, *n* = 6) nmol * OD_750_^−1^ * mL^−1^ min^−1^ and a A*^13^C*_2,3-C2_ equal to 0.23 ± 0.02 (SE, *n* = 6) nmol * OD_750_^−1^ * mL^−1^ min^−1^ (Supplementary Table S6). Whereas, *Δgapdh1* did not differ from WT, *Δgapdh2* assimilated with lower A*^13^C*_1-C_ rate equal to 0.03 ± 0.01 (SE, *n* = 6) nmol * OD_750_^−1^ * mL^−1^ min^−1^.

As a consistency check and to characterize the differential time-lag of ^13^C incorporation, we monitored the midpoint time of the fitted logistic sigmoidal functions (Fig. 8D to F). We expected a lag between the CBB-cycle and RUBISCO activities. In agreement with this expectation, A*^13^C*_1-C_ midpoint times of WT and the *Δgapdh1* mutant were 5.4 ± 0.4 and 6.1 ± 0.6 (SE, *n* = 6) min, significantly earlier than the respective A*^13^C*_2,3-C2_ midpoint times at 8.4 ± 0.4 and 8.8 ± 0.6 (SE, *n* = 6) min. The average A*^13^C*_1-C_ midpoint time of the *Δgapdh2* differed significantly between HC- and LC-preacclimation (Fig. 8F; Supplementary Table S6). The A*^13^C*_1-C_ midpoint was similar to WT after LC-preacclimation but ∼1.9-fold delayed after HC preacclimation. The A*^13^C*_2,3-C2_ midpoint time of the *Δgapdh2* mutant was not detectable in the absence of ^13^C incorporation into 2,3-C_2_ of 3PGA.

## Discussion

Carbon positional analyses are typically the domain of ^13^C-nuclear magnetic resonance (NMR) analyses (Hoffman and Rasmussen 2019). Our method is based on routine GC-MS technology for the profiling of primary metabolism (Fiehn et al. 2000; Lisec et al. 2006). It can be applied widely to small and complex biological samples that may be hard to analyze by ^13^C-NMR. We exploit *in source* fragmentation of GC-MS technologies to calculate positional labeling information (Wittemeier et al. 2024). This procedure is delimited to fragmentation reactions that are substance specific and in part depend on the choice of mass spectral ionization technologies, e.g. GC-EI-(TOF)MS or GC-APCI-(TOF)MS (Fig. 1). Secondary MS-MS or MS^n^ inducible fragmentation technologies have been applied to other metabolites, e.g. (Choi et al. 2012 ), and remain to be explored for refinement of 3PGA analyses. We established a method that enables in vivo C-positional measurements of molar ^13^C assimilation into carbon atom 1-C of 3PGA and into carbon atoms 2,3-C_2_ of the same molecule. ^13^C assimilation into position 1-C of 3PGA monitors in vivo RUBISCO activity. We directly monitor molar ^13^C assimilation, but our method does not inform which factors, e.g. RUBISCO amount, substrate availability or metabolic regulation, may be causal.

We chose 5.0% ^13^CO_2_ (HC) for pulse labeling to investigate a physiological state at which the highly active *Synechocystis* RUBISCO (Marcus et al. 2005, 2011) is likely not limiting. The time lag between A*^13^C*_1-C_ and A*^13^C*_2,3-C2_ (Fig. 8E and F) in combination with initial nonhomogenous labeling of 3PGA carbon-positions (Fig. 3) demonstrated this assumption. ^13^C assimilation into 2,3-C_2_ assesses the activity of carbon-cycling through the CBB reactions in combination with anaplerotic reactions that supplement and stabilize the CBB cycle (Makowka et al. 2020). Under photosynthetic pulse labeling conditions, the anaplerotic PHOSPHOGLUCOISOMERASE (PGI) and OPP shunts can provide additional nonlabeled carbon from previously generated storage carbohydrates to regenerate RuBP (Makowka et al. 2020). The relative contribution of anaplerosis becomes apparent at saturating ^13^C incorporation under steady-state labeling conditions of 3PGA that are approximated at 60–90 min in our experimental setup (Fig. 3B to E). As was expected from known high glycogen accumulation under HC conditions compared to lower glycogen levels in LC cells (Eisenhut et al. 2007), HC preacclimated cells had consistently more anaplerotic contribution to 3PGA synthesis as evidenced by lower E*^13^C*_1,2,3-C3_ and E*^13^C*_2,3-C2_ (Fig. 3B and C) at 90 min of the pulse but almost equal E*^13^C*_1-C_ (Fig. 3D) and consequently lower E*^13^C*_2,3-C2/1-C_ (%) (Fig. 3E).

In this study, we focused on measurements of ^13^C assimilation rates through dynamic photosynthetic labeling experiments. Next to the steady-state condition, HC-HC, we included a nonsteady-state LC-HC shift. A correction for fluctuating 3PGA concentrations during preacclimation and pulse labeling and to compare between different mutants is necessary to avoid potential misinterpretations of E*^13^C* observations. Our in vivo method extends the current photosynthetic phenotyping portfolio of methods that monitor RUBISCO activity. Our technology provides direct C-positional flux information. Thereby, we generate additional constraints for metabolic modeling based on carbon fate maps of metabolism. The validation of our methodology was complicated by nonavailability of C-position labeled 3PGA reference substances. We decided to validate position specificity of our method by in vivo metabolization of commercially available positional labeled glucose isotopomers. We did not use in vitro biosynthesis of 3PGA from glucose substrate, because required active enzyme preparations that were available to us always contained nonlabeled metabolic substrates or products. These impurities were present at varying amounts and confounded an in vitro approach to prove position specificity. The metabolization of 3,4-^13^C_2_-glucose by *E. coli* K-12 strains compared to control substrates indicated the positional specificity of 3PGA labeling assuming preferred metabolization of glucose through the EMP pathway (Fig. 2; Supplementary Fig. S1). But we observed redistribution of ^13^C from 3,4-^13^C_2_-glucose by *E. coli* K-12 into carbons 2,3-C_2_ of 3PGA. These observations agreed with expected minor OPP pathway activity (Hollinshead et al. 2016). We demonstrated positional specificity of our method in the absence of an active OPP pathway using *Δzwf* and *Δgnd* mutants of *E. coli* (Fig. 2D). Additional prove of 1-C position selectivity of our method came with the discovery that the *Δgapdh2* mutant of *Synechocystis* incorporates ^13^CO_2_ exclusively into 1-C of 3PGA in agreement with the known reaction mechanism of RUBISCO (Fig. 3A), while 2,3-C_2_ of 3PGA remained nonlabeled (Fig. 6C, F, and I; Supplementary Table S5) due to the interrupted CBB cycle in the mutant (Schulze et al. 2022).

We validated E*^13^C* and C*^13^C* quantifications through two GC-(TOF)MS technologies by exploration of instrument characteristics, potential analytical interferences, and analyses of the linear range of abundance quantifications. Besides our paired mode of 3PGA abundance quantification, any technology that is not affected by isotope labeling can be used to determine C_3PGA_ and resulting C*^13^C*. We demonstrated the later by comparing NIA-corrected abundance sums of differentially labeled isotopologue distributions to abundance measurements that use mass features of 3PGA that do not receive ^13^C label (Supplementary Table S1). Both approaches were equivalent (Supplementary Table S4). E*^13^C* quantifications depend on accurate quantification of mass isotopologue distributions that can be subject to mass spectrometric instrument bias as we demonstrated by comparison of our GC-(TOF)MS instruments (Fig. 5). Results from the two instruments were not exactly equivalent. Our GC-EI-(TOF)MS performed better for C_3PGA_ and resulting C*^13^C* quantifications, whereas the high mass resolution GC-APCI-(TOF)MS instrument was superior for E*^13^C* quantifications. We demonstrated that E*^13^C* quantifications are confounded at low metabolite concentrations and by saturation at upper metabolite detection limits and only valid within tested metabolite concentration ranges (Fig. 4A and C). In addition, isobaric interference needs to be tested and avoided to obtain biologically meaningful E*^13^C* data. In the absence of ^13^C-labeled 3PGA reference substance, we resorted to E*^13^C* interference analysis using nonlabeled 3PGA. E*^13^C* analysis of nonlabeled 3PGA is based on the expectation that NIA-correction must adjust E*^13^C* of measured natural isotopologue distributions of all molecular features with known molecular formula to zero. This test was highly efficient when applied to chemically pure reference compounds or complex mixtures. It detects mass features from the same compound or coeluting compounds that interfere with isotopologue distributions of interest and may detect potential misinterpretations of molecular formulas.

Due to the mass shift of labeled isotopologue distributions, similar tests are advised at full ^13^C labeling if a certified reference compound is available as exemplified in this study by labeled sorbitol and glucose isotopomers (Supplementary Table S3). To account for potential interferences or instrument bias at high E*^13^C* of 3PGA, we analyzed the paired set of 3PGA measurements of our GC-EI-(TOF)MS and GC-APCI-(TOF)MS instruments. In the absence of labeled 3PGA reference substance with precisely defined isotopic purity, we cannot directly prove which instrument is more accurate for E*^13^C* measurements. But with GC-APCI-(TOF)MS, we chose the technology that provided the more plausible E*^13^C* data. In addition, a high mass resolution technology is inherently less prone to isobaric interferences. The most decisive criterion for the choice of GC-APCI-(TOF)MS for E*^13^C* determination from this study was that GC-APCI-(TOF)MS data met the expectation that E*^13^C*_2.3-C2/1-C_ (%) cannot exceed 100% during photosynthetic pulse labeling experiments (Fig. 5D).

Our technology can be transferred to other organisms, metabolites and mass spectrometric instrumentation. Ionization technologies of mass spectrometers that induce a suitable set of fragments and molecular ions are prerequisite for combinatorial calculations of positional E*^13^C* or direct measurements of single carbon-positions, e.g. 2-C or 4-C of malic acid (Okahashi et al. 2019). In each new application case, the mass spectrometric technology should be assessed for accurate representation of isotopologue distributions ideally in combination with accurate performance of concentration measurements to enable molar C*^13^C* and *A^13^C* measurements under fluctuating conditions. When using a different biological system, the altered metabolite concentrations of a different metabolic state, will affect analytical sensitivity and interferences. Importantly the timing until E*^13^C* saturation will differ and result in the need to adjust sampling speed and frequency for dynamic pulse labeling. For example, a biological system with low 3PGA concentrations and high RUBISCO enzyme activities will reach ^13^C saturation of 3PGA faster and vice versa. The transfer to other metabolites may be constrained by specific analytical interferences, sensitivity, or abundance saturation issues. Most importantly, C-positional analysis will be constrained by the available compound specific *in source* or induced fragmentation reactions, e.g. (Young et al. 2011; Okahashi et al. 2019; Lima et al. 2021; Wittemeier et al. 2024).

Our study included application cases to demonstrate that important insights can be gained from our technology. We analyzed the effect of HC versus LC preacclimation on carbon assimilation under HC conditions in combination with the function of GAPDH in *Synechocystis,* where GAPDH2 is required for photoautotrophic growth and the role of GAPDH1 is enigmatic (Koksharova et al. 1998; Schulze et al. 2022). We demonstrate by careful correction for changes of C_3PGA_ that the differential preacclimation does not affect carbon assimilation rates A*^13^C* (nmol * OD_750_^−1^ * mL^−1^ * min^−1^) into 1-C, 2,3-C_2_ or the complete 3PGA molecule when using a high ^13^CO_2_ pulse (Fig. 8A to C; Supplementary Table S6). Likely the high availability of external CO_2_ and its fast diffusion toward RUBISCO overrides the effect of a Ci concentration mechanism in combination with deactivation of the CCM upon shift from low to high Ci.

Glyceraldehyde-3-phosphate dehydrogenation is thought to be a central reaction step that enters newly assimilated carbon from 3PGA into the CBB cycle and upper carbon metabolism toward glycogen synthesis. The two GAPDH enzyme isoforms of *Synechocystis* are highly divergent (Figge et al. 1999) and clearly have different functions, where GAPDH2 is strictly required for photoautotrophic growth due to its ability to use NADPH in anabolic direction (Koksharova et al. 1998; Schulze et al. 2022). The *Δgapdh2* mutant of *Synechocystis* is thought to be CBB cycle deficient and is not viable without provision of an external organic carbon source, such as glucose. The function of GAPDH1, which cannot utilize NADP or NADPH, remains enigmatic as the *Δgapdh1* mutant of *Synechocystis* is fully viable and did not show an obvious phenotype. GAPDH1 is thought to function exclusively in glycolytic direction of the GAPDH reaction. In contrast, GAPDH2 appears to function bidirectionally and can be expected to be sufficient for both, glycolysis and the CBB cycle.

We discovered that the *Δgapdh1* mutant is not capable of rapid readjustment of the 3PGA concentration after LC-HC shift (Fig. 6B). After long-term HC acclimation *Δgapdh1*, however, adjusts to lower 3PGA concentrations and does not differ from WT in our HC-HC experiments (Fig. 6; Supplementary Table S6). This finding suggests that GAPDH1 activity is involved in the rapid readjustment of 3PGA concentrations upon fluctuations, e.g. of Ci availability. The increased E*^13^C*_2.3-C2/1-C_ (%) of *Δgapdh1* relative to WT, specific for LC preacclimated cells (Fig. 7B), indicates a lower relative contribution of anaplerotic carbon provision in agreement with the proposed catabolic role of GAPDH1. In addition, the marginal increase of A*^13^C*_2,3-C2_ (nmol * OD_750_^−1^ * mL^−1^ * min^−1^) in *Δgapdh1* relative to WT (Fig. 8B) may indicate a minor, LC-specific, catabolic activity of GAPDH1 that counteracts the anabolic 3-phosphoglyceraldehyde production catalyzed by GAPDH2. It has been shown that its activity is influenced by the regulatory protein CP12 under fluctuating Ci conditions (Lucius et al. 2022), while the activity of GAPDH1 is not affected by this regulatory switch. Constitutive activity of such a slightly wasteful but balanced GAPDH1-GAPDH2 reaction system may come at the benefit of high-speed rebalancing between anabolic and catabolic directions that should be required to respond to rapid environmental fluctuations.

The analyses of the *Δgapdh2* mutant revealed clear insights in its essential function within the CBB cycle. The absence of ^13^C incorporation into 2,3-C_2_ of 3PGA in the *Δgapdh2* mutant proves that GAPDH1 alone is not sufficient to sustain the CBB cycle in *Synechocystis* as was proposed before in the genetic approach (Figs. 6 and 8; Supplementary Table S5) (Koksharova et al. 1998; Schulze et al. 2022). Surprisingly, we discovered ^13^C incorporation into the full 3PGA molecule and confirmed unequivocally that this incorporation is exclusive to the 1-C position (Fig. 6F) with assimilation rates A*^13^C*_1-C_ (nmol * OD_750_^−1^ * mL^−1^ * min^−1^) of *Δgapdh2* amounting to 12% (HC-HC) or 27% (LC-HC) of the WT (Fig. 8C; Supplementary Table S6). Assimilation of ^13^CO_2_ in *Δgapdh2* was measured in the absence of added glucose during the ^13^CO_2_ pulse and, consequently, must depend on catabolism of storage carbohydrate that accumulated during the preacclimation phase in the presence of nonlabeled glucose. The glycolytic PGI and OPP shunts that likely utilize the glycogen pool replenish the CBB cycle intermediates in the absence of GAPDH2 as was suggested (Makowka et al. 2020) and allow CO_2_ fixation via RUBISCO in the absence of a fully functional CBB cycle. This observation allows us to propose that *Synechocystis* can operate a catabolic pathway that includes RUBISCO activity. Using the known anaplerotic shunts (Makowka et al. 2020), RUBISCO can support a glycolytic route composed of the decarboxylating, oxidative part of the OPP pathway for RuBP production and RUBISCO activity that re-assimilates CO_2_ that is lost by oxidative decarboxylation of the 6-phosphogluconate dehydrogenase reaction step or by other cellular decarboxylating reactions. A similar role of RUBISCO has been proposed to exist in developing embryos of *Brassica napus L.* (oilseed rape) (Schwender et al. 2004). RUBISCO was shown to operate without the CBB cycle in a function that optimizes efficient storage lipid accumulation. Compared to glycolysis, this RUBISCO pathway was estimated to generate more acetyl-CoA for fatty acid biosynthesis and, importantly, looses 40% less carbon as CO_2_ (Schwender et al. 2004). Re-assimilation of CO_2_ that is released by pyruvate decarboxylase, the OPP pathway and the TCA cycle is thought to increase the efficiency of carbon use in oilseed rape embryos (Schwender et al. 2004). In cyanobacteria, the maintenance of a CO_2_ scavenging path that likely evolved before RUBISCO was recruited for photosynthetic carbon assimilation (Aono et al. 2015; Schönheit et al. 2016; Erb and Zarzycki 2018) seems plausible. During early cyanobacteria phylogeny, a high CO_2_ environment was prevalent before the oxygenation of Earth's atmosphere. CO_2_ should have been abundantly available for scavenging with the potential advantage of minimizing the loss of Ci through catabolic physiological phases. Whether the proposed catabolic OPP-RUBISCO path that bypasses glyceraldehyde-3-phosphate and the carbohydrate phosphates of the EMP, OPP, and CBB pathways is active in recent *Synechocystis* WT or an atavism revealed by the *Δgapdh2* mutation, remains to be investigated.

In conclusion, we developed and validated a minimally invasive methodology for in vivo RUBISCO activity measurement, combined with a proxy for CBB cycle activity, by carbon-positional measurements of 3PGA using *in source* fragmentation reactions inherent to GC-MS technology. We applied our methodology to study *Synechocystis* metabolism and revealed evidence of a catabolic pathway involving RUBISCO activity without the CBB cycle. Orthogonally, we explored the role of GAPDH1 under Ci shift conditions and propose a function for this enzyme. The results may spark additional directions of research on in vivo RUBISCO activity and its potential role without a CBB cycle.

## Materials and methods

### Cyanobacteria cultivation, and ^13^CO_2_ labeling experiments

The glucose tolerant *Synechocystis sp.* PCC 6803 wild-type strain of this study was provided by N. Murata (National Institute for Basic Biology, Japan). The corresponding *Δgapdh2* mutant of GAPDH2 (*sll1342*) was previously described (Schulze et al. 2022). The *Δgapdh1* mutant, lacking GAPDH1 (*slr0884*) was constructed in two subsequent steps using the workflow described by (Chen et al. 2016). In short, deletion constructs were assembled from a chloramphenicol resistance cassette and from 200 bp long flanking regions upstream and downstream of the target gene using Gibson assembly and cloned into pBluescript with details described earlier (Chen et al. 2016). *Synechocystis sp.* PCC 6803 was then transformed with the plasmid. The mutants were checked by Southern blotting for segregation and the correct genotype (Supplementary Fig. S4A) with corresponding primers (Supplementary Fig. S4B). Analogous mutants, namely *Δgap1^−^*, i.e. *Δgapdh1*, and *Δgap2^−^*, i.e. *Δgapdh2*, were previously characterized (Koksharova et al. 1998). *Synechocystis* cells were cultivated under continuous illumination at 100 µmol photons m^−2^ s^−1^ in a multicultivator MC 1000-OD photobioreactor (Photon Systems Instruments, Drásov, Czech Republic) using BG-11 medium (Rippka et al. 1979). The medium was buffered at pH 8 by 20 mM TES-KOH and bubbled at a flow rate of approximately two bubbles per second with high (5%, v/v) CO_2_ enriched air (defined as HC condition); low CO_2_ preacclimation was by bubbling ambient air, ∼0.04% CO_2_, at the same rate with BG-11 medium adjusted to pH 7 (defined as LC condition) (Orf et al. 2015). Replicate cultures of WT and mutants were randomized across the 8 cultivation positions of the photobioreactor. Replicate experiments with the photobioreactor were preacclimated either to LC or the HC conditions. Initial cultures were cultivated for at least four days at 30 °C and grown to approximately equal optical density at wave length 750 nm (OD_750_), OD_750_ ∼ 1.2 (HC) or OD_750_ ∼ 0.6–1.00 (LC). Cells were transferred to fresh medium ∼ 4 h before the ^13^CO_2_ pulse experiment and continued under preacclimation conditions. First samplings at t_0_ were harvested from the HC or LC preacclimated cultures. Subsequently, dissolved nonlabeled CO_2_ was removed by fast medium exchange with continuous illumination. Bubbling was immediately resumed with 5% ^13^CO_2_ in artificial air. Sample volumes equivalent to ∼ 10.0 OD_750_ * mL were collected with continued illumination and immediately shock frozen in liquid N_2_ at 5, 10, 15, 30 and 60 min after onset of ^13^CO_2_ bubbling. The exact OD_750_ * mL equivalent of each sample was used as reference to calculate molar concentrations. A 90 min sample was taken for E*^13^C* analyses but due to volume restrictions did not allow paired measurements of 3PGA concentration of all replicates. Medium exchanges and samplings were by fast (<15 s) vacuum filtration onto PVDF membrane filters (0.45 µm pore size) or glass fiber filters (1.2 µm pore size), respectively, with continuous illumination (Huege et al. 2011; Orf et al. 2015). The *Δgapdh2* mutant was cultivated and preacclimated in the presence of 55 mM glucose in BG-11 medium (Koksharova et al. 1998). Glucose was removed from *Δgapdh2* cultures with the last medium exchange. The 5% ^13^CO_2_-pulse was in the absence of an external organic carbon source. *M. aeruginosa* PCC 7806 was cultivated for three days at 20 °C in BG-11 medium until OD_750_ ∼ 0.9 was reached. 10 mL of culture were harvested by fast vacuum filtration using glass fiber filters (1.6 µm pore size).

### *E. coli* cultivation, and ^13^C-glucose labeling experiments

*E. coli* strain K-12 MG1655 was cultivated at 28 °C in chemically defined M9 mineral minimal medium using glucose (10 mM) as sole carbon source (Paliy and Gunasekera 2007). Precultures were split into replicates cultures and OD_600_ adjusted to ∼2.0. 5 mM ^13^C-labeled glucoses were added by rapid medium exchange. Sample volumes amounting to ∼20 OD_600_ * mL equivalents were harvested after 90 min. Control samples cultivated with nonlabeled glucose were harvested at t_0_ immediately before the ^13^C-glucose pulses. Rapid medium exchange and sampling were by 5 min centrifugation at ∼12,000 × *g* and 28 °C. Samples were shock frozen after thorough aspiration of the centrifugation supernatant. Nonlabeled glucose and each glucose isotopomer were tested by two independent cultivation experiments. *E. coli* K-12 *Δzwf* and *Δgnd* mutants defective for the OPP pathway and the knock-out parent strain BW25113 were from the “Keio” single-gene knockout mutant collection (Baba et al. 2006). Glycerol stocks, OEC5042 (parent strain), and two independent knock-out clones per gene, OEC4987-213603781 (*Δzwf-1*, JW1841), OEC4987-200827214 (*Δzwf-2*, JW1841), OEC4987-213603783 (*Δgnd-1*, JW2011), and OEC4987-200827363 (*Δgnd-2*, JW2011) were obtained from Horizon Discovery Biosciences Limited (Cambridge, UK) and cultivated as described above.

### Reference chemicals

The stable isotope labeled precursor chemicals and reference chemicals of this study were: ^13^CO_2_ (99.0 atom % ^13^C, Sigma-Aldrich, 364592), ^13^C_6_-glucose, i.e. [U-^13^C]-glucose (≥99 atom % ^13^C, ≥99% chemical purity; Sigma-Aldrich, 389374), 1,2-^13^C_2_-glucose (≥99 atom % ^13^C; Sigma-Aldrich 453188), 3,4-^13^C_2_-glucose (Omicron Biochemicals), 1,6-^13^C_2_-glucose (≥99 atom % ^13^C, 99% chemical purity; Sigma-Aldrich, 453196), 1-^13^C_1_-glucose (≥99 atom % ^13^C; Sigma-Aldrich 297046), 2-^13^C_1_-glucose (≥99 atom % ^13^C; Sigma-Aldrich 310794), 3-^13^C_1_-glucose (≥99 atom % ^13^C; 99% chemical purity; Sigma-Aldrich 605409), 4-^13^C_1_-glucose (≥99 atom % ^13^C; Sigma-Aldrich 668648), 5-^13^C_1_-glucose (≥98 atom % ^13^C; 98% chemical purity; Sigma-Aldrich 717355), 6-^13^C_1_-glucose (≥99 atom % ^13^C; Sigma-Aldrich 310808), the 3PGA standard substance for quantitative calibration (≥93% dry basis (enzymatic); Sigma-Aldrich, P8877), and internal standard ^13^C_6_-sorbitol (99 atom % ^13^C, 99% chemical purity; Sigma-Aldrich, 605514).

### Metabolite extraction and chemical derivatization

Polar metabolites were extracted from deep frozen cells on filters, as described before (Erban et al. 2020) by adding 1 mL of an extraction mixture consisting of methanol (≥99.9% gradient grade for liquid chromatography, Sigma-Aldrich), chloroform (≥99.8%, ACS reagent grade, contains ethanol stabilizer; Sigma-Aldrich), purified water (Milli-Q Typ-1-Reinstwassersysteme, Merck KGaA, Darmstadt, Germany) in a ratio of 2.5:1:1 (v/v/v), and 6 µg * mL^−1^ of ^13^C_6_-sorbitol for quantitative internal standardization. Samples were incubated at 70 °C for 15 min. An aqueous phase was separated by adding 400 µL of water to the extracts and centrifuging at ∼12,000 × *g* for 10 min. The upper aqueous phase, 800–1200 µL, was dried by vacuum centrifugation overnight. The dried metabolite extracts and quality control samples were subjected to methoxyamination and trimethylsilylation as previously described (Fiehn et al. 2000). An alkane mixture comprising C_10_, C_12_, C_15_, C_18_, C_19_, C_22_, C_28_, C_32_, and C_36_ n-alkanes was added to the samples for retention index calculation (Strehmel et al. 2008).

### Gas chromatography-mass spectrometry

Derivatized samples were analyzed by an Agilent 6890N24 gas chromatograph (Agilent Technolo-gies, Waldbronn, Germany) hyphenated to either EI-time of flight-mass spectrometry, GC-EI-(TOF)MS, using a LECO Pegasus III time of flight mass spectrometer (LECO Instrumente GmbH, Mönchengladbach, Germany) or to atmospheric pressure chemical ionization-time of flight-mass spectrometry, GC-APCI-(TOF)MS, with a micrOTOF-Q II hybrid quadrupole time-of-flight mass spectrometer (Bruker Daltonics, Bremen, Germany) equipped with an APCI ion source and GC interface (Bruker Daltonics) (Kopka et al. 2017; Wittemeier et al. 2024). All measurements were conducted in splitless mode using 5% phenyl—95% dimethylpolysiloxane fused silica capillary columns with 30 m length, 0.25 mm inner diameter, 0.25 µm film thickness, and an integrated 10 m precolumn (Agilent Technologies, CP9013) (Erban et al. 2020).

### Chromatography data processing and metabolite annotation

GC-EI-(TOF)MS chromatograms were exported as netCDF files after baseline correction and smoothing using ChromaTOF software (version 4.22, LECO) as previously described (Erban et al. 2020). The processed chromatograms were then subjected to combined chromatography data analysis at nominal mass resolution using TagFinder (Luedemann et al. 2008, 2012), the NIST MS Search 2.0 software (http://chemdata.nist.gov/), and the R package XLConnect: Excel Connector for R (version 1.0.7; https://CRAN.R-project.org/package=XLConnect) in RStudio (2023.6.1.524, R version 4.3.1; http://www.posit.co/) to extract peak apex abundances corresponding to the isotopologues of molecular and fragment ions of interest. Analytes were annotated by matching retention indices and mass spectra from nonlabeled samples to the data of reference compounds from the GMD (Kopka et al. 2005).

GC-APCI-MS files were internally mass-calibrated by perfluorotributylamine (PFTBA, FC43). The chromatograms were exported in mzXML format using DataAnalysis and AutomationEngine software (version 4.2; Bruker Daltonics, Bremen, Germany). Analytes within GC-APCI-(TOF)MS files were identified manually, based on expected exact monoisotopic m/z, retention time, and mass spectrum comparisons to paired GC-EI-(TOF)MS analyses (Wittemeier et al. 2024) and paralleled measurements of 3PGA, glucose and sorbitol reference compounds. The isotopologue abundances of molecular and fragment ions and respective natural or ^13^C labeled mass isotopologue distributions were extracted from each GC-APCI-(TOF)MS file within a defined chromatographic time range. This time range was manually adjusted to each analyte and a m/z range of ±0.005 units was applied throughout. Exact monoisotopic and isotopologue abundances were extracted using the R packages XCMS (version 3.22.0) (Tautenhahn et al. 2008), MSnbase (version 2.26.0) (Gatto et al. 2021), and msdata (version 0.40.0; https://www.bioconductor.org/packages/release/data/experiment/html/msdata.html) in RStudio. Quantification of abundances was by area under the chromatographic peak.

### ^13^C Enrichment analysis

The extracted isotopologue abundances of mass features, i.e. molecular, adduct, and fragment ions, were processed by the R package IsoCorrector (version 1.18.0) (Heinrich et al. 2018) to quantify the ^13^C fractional enrichment (E*^13^C*), obtain isotopologue distributions corrected for the NIA of elements, the sum of NIA-corrected isotopologues of each mass feature and their relative isotopologue abundance (RIA) distributions.

### Concentration analysis of 3PGA

The concentration of 3PGA was determined using the sum of NIA-corrected isotopologue abundances from nonlabeled and labeled samples. The sums of NIA-corrected isotopologue abundances were normalized to internal standard ^13^C_6_-Sorbitol, OD_750_ and sample volume. Molar concentrations of 3PGA (C_3PGA_) were acquired through parallel analysis of calibration series of nonlabeled 3PGA reference compound. E*^13^C* and C_3PGA_ were multiplied to calculate molar ^13^C concentrations (C*^13^C*) of the complete molecule and at specific carbon positions. Further calculations are reported in the results section.

### Statistical analyses and curve fitting

Carbon assimilation rates into 1-C of 3PGA were determined by sigmoidal curve fitting using R Studio and the package sicegar (version 0.2.4) (Caglar et al. 2018) with default settings and 1,000 iterations to test the significance of the sigmoidal fit. C*^13^C*, i.e. pmol × OD_750_^−1^ × mL^−1^, at 0, 5, 10, 15, 30, and 60 min served as input data for curve fitting. The threshold intensity ratio was 0.75 and the maximum allowed intensity at t_0_ set to 0. The maximum slope from the sigmoidal equations was defined as the maximum assimilation rate (A^13^C) in units of pmol × OD_750_^−1^ × mL^−1^ × min^−1^. The time (min) at the maximum assimilation rates from the sigmoidal equations was recorded to assess the delay of ^13^C dilution relative to the pulse. Initial carbon assimilation rates after exponential regression were estimated using the statistic function “(nls())” of R Studio software. We calculated the first derivative k * I_max_ at t_0_ of I_max_(1-e^−kt^), where t is time after pulse, k is the kinetic rate constant, and I_max_ is the maximum of the function. The Student’s *t*-tests and Pearson’s correlation analyses were performed using R Studio or Microsoft Excel^TM^ functions.

### Accession numbers

*Synechocystis* sp. PCC 6803 genes *GAPDH1* and *GAPDH2* have ordered locus names slr0884 and sll1342, respectively. *E. coli* genes *ZWF* and *GND* have ordered locus names b1852 (JW1841) and b2029 (JW2011).

## Supplementary Material

kiaf020_Supplementary_Data

## Data Availability

The data underlying this article are available in the article and in its online supplementary material.

## Supplementary data

The following materials are available in the online version of this article.

**Supplementary Figure S1.** Carbon position mapping between glucose-6-phosphate and 3PGA through the Embden-Meyerhof-Parnas (EMP) pathway.

**Supplementary Figure S2.** Carbon position mapping between glucose and 3PGA through the oxidative pentose phosphate (OPP) pathway.

**Supplementary Figure S3.** C assimilation rates (A*^13^C*) into positions 1,2,3-C_3_, 2,3-C_2_, and 1-C of 3PGA from high CO_2_ (HC, 5.0%) and low CO_2_ (LC, ambient) preacclimated wild-type *Synechocystis sp.* PCC 6803 compared to Δ*gapdh1* and Δ*gapdh2* mutant cells.

**Supplementary Figure S4.** Molecular characterization of the *Δgapdh1* deletion mutant.

**Supplementary Table S1.** Validated in silico fragmentation analysis of 3-PGA (4TMS) analyzed by GC-EI(TOF)-MS and GC-APCI(TOF)-MS.

**Supplementary Table S2.** NIA-corrected E*^13^C* of chemically pure nonlabeled 3PGA reference substance compared to E*^13^C* of 3PGA from complex extracts of nonlabeled *Synechocystis sp.* PCC 6803 or *Microcystis aeruginosa* PCC7806.

**Supplementary Table S3.** Accuracy and precision of E*^13^C* measurements by GC-APCI-(TOF)MS using certified chemical reference substances.

**Supplementary Table S4.** Relative standard deviations (RSD) of 3PGA quantifications by GC-EI-MS using either sums of NIA-corrected isotopologue abundances or monoisotopic mass fragments that did not incorporate ^13^C.

**Supplementary Table S5.** 3PGA concentration and molar carbon assimilation analysis into positions 1-C, 2,3-C_2_ and 1,2,3-C_3_ of 3PGA of high CO_2_ (HC, 5.0%) and low CO_2_ (LC, ambient) preacclimated wild type, *Δgapdh1* and *Δgapdh2* mutant cells of *Synechocystis sp.* PCC 6803.

**Supplementary Table S6.** Assimilation rates of ^13^C into 1,2,3-C_3_, 2,3-C_2_, and 1-C of 3PGA, estimated by exponential or logistic sigmoidal fitting of dynamic molar ^13^CO_2_ assimilation kinetics of high CO_2_ (HC, 5.0%) and low CO_2_ (LC, ambient) preacclimated wild type, *Δgapdh1* and *Δgapdh2* mutant cells of *Synechocystis sp.* PCC 6803.

## Dive Curated Terms

The following phenotypic, genotypic, and functional terms are of significance to the work described in this paper:

carbon CHEBI: CHEBI:27594

NADP CHEBI: CHEBI:25523
