# Supplementary material for: Dynamic photosynthetic labeling and carbon-positional mass spectrometry monitor in vivo RUBISCO carbon assimilation rates
Source: Plant Physiol. 2025 Jan 21;197(2):kiaf020. doi: 10.1093/plphys/kiaf020 (PMC11809591; doi:10.1093/plphys/kiaf020)
Supplement: kiaf020_Supplementary_Data [file kiaf020_supplementary_data.pdf]

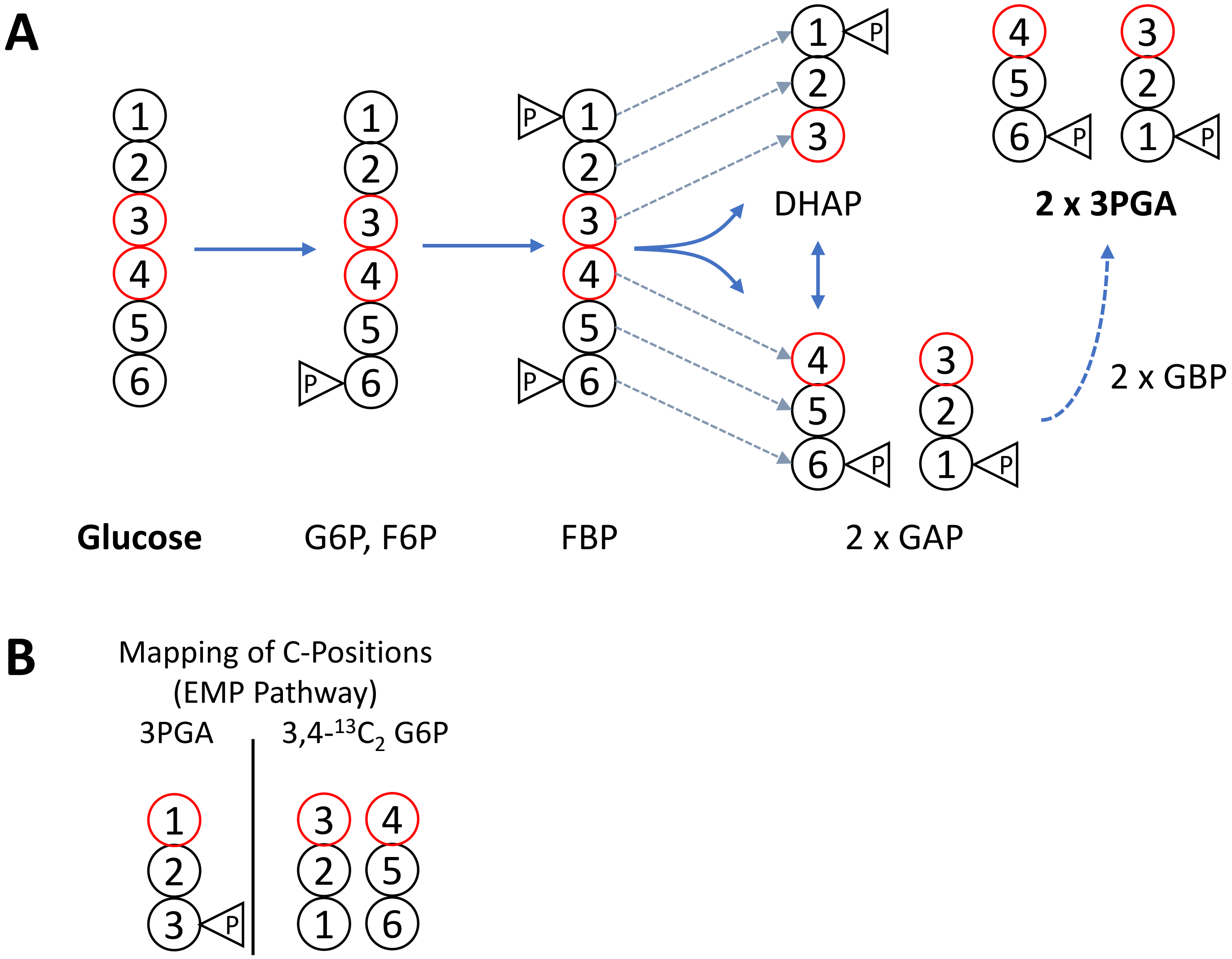

A
Glucose
G6P, F6P
FBP
DHAP
2 x GAP
2 x GBP
2 x 3PGA
B
Mapping of C-Positions
(EMP Pathway)
3PGA
3,4-$^{13}C_2$ G6P

Supplementary Figure S1. Carbon position mapping between glucose-6-phosphate and 3PGA through the Embden-Meyerhof-Parnas (EMP) pathway.

(A) The EMP pathway converts one molecule of glucose into two molecules of 3PGA. The carbon configuration is maintained between glucose and FBP. FBP aldolase (EC 4.1.2.13) cleaves the carbon bond between 3-C and 4-C of FBP.

(B) Carbon positions 1-C, 2-C, and 3-C of 3PGA generated through the EMP pathway originate from 3-C and 4-C, 2-C and 5-C, and 1-C and 6-C of glucose, respectively.

Glucose-6-phosphate (G6P), fructose-6-phosphate (F6P), fructose-1,6-bisphosphate (FBP), dihydroxyacetonephosphate (DHAP), glyceraldehyde-3-phosphate (GAP), 1,3-bisphosphoglyceric acid (GBP), 3-phosphoglyceric acid (3PGA). Red outline indicates mapping between 1-C of 3PGA and 3-C or 4-C of G6P. Dashed grey arrows indicate the C-mapping of the FBP aldolase reaction. The dashed blue arrow indicates more than 1 reaction.

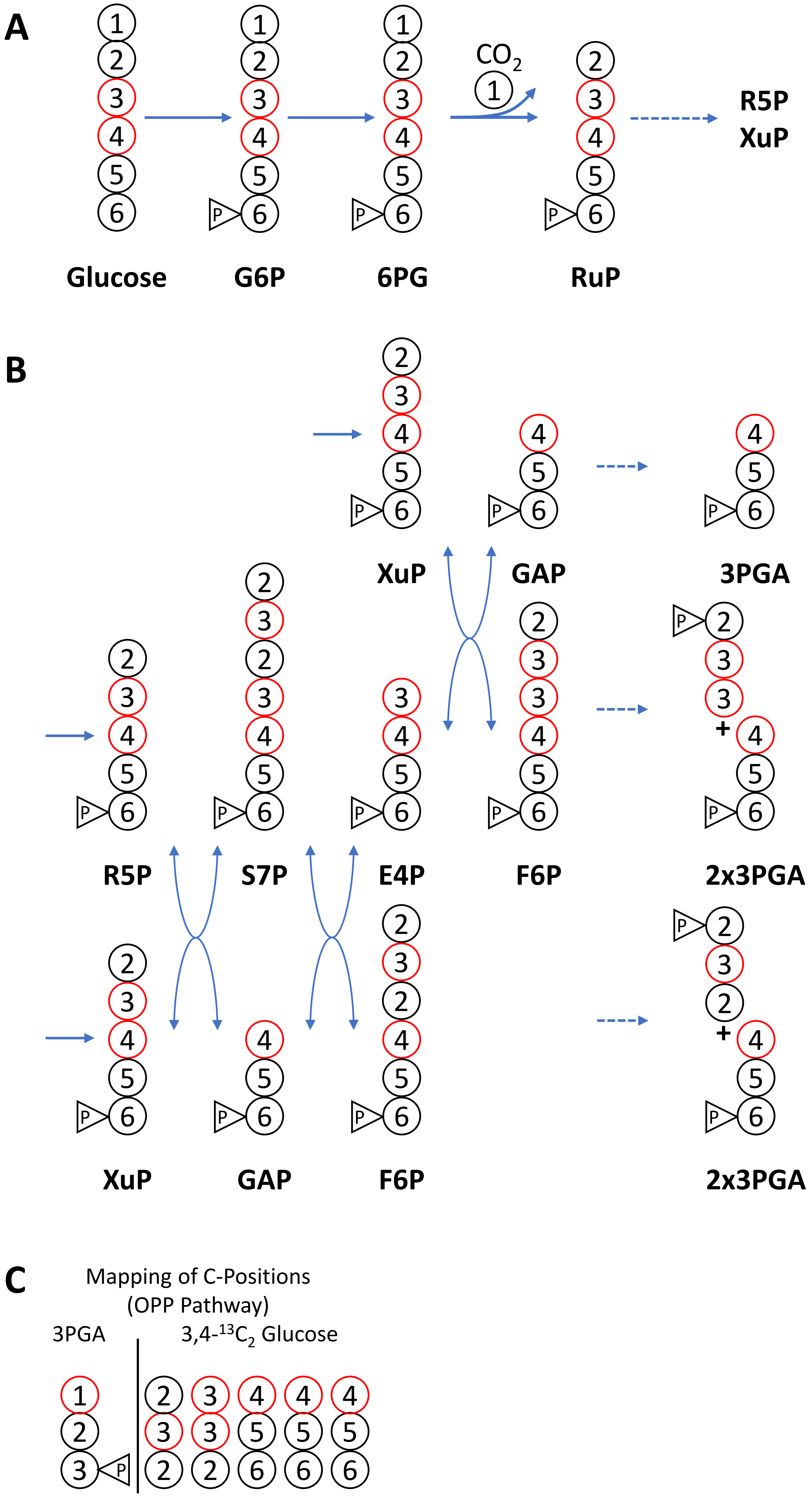
A
Glucose
G6P
6PG
CO_2
RuP
R5P
XuP
B
XuP
GAP
3PGA
R5P
S7P
E4P
F6P
2x3PGA
XuP
GAP
F6P
2x3PGA
C
Mapping of C-Positions
(OPP Pathway)
3PGA
3,4-$^{13}C_2$ Glucose

Supplementary Figure S2. Carbon position mapping between glucose and 3PGA through the oxidative pentose phosphate (OPP) pathway.

(A) The OPP pathway converts 3 molecules of glucose into 5 molecules of 3PGA and decarboxylates 1-C of glucose via 6PG dehydrogenase.

(B) The carbon configuration of the resulting pentoses is rearranged by sequential transketolase (EC 2.2.1.1) and transaldolase (EC 2.2.1.2) reactions that rearrange 2-C and 3-C derived carbon atoms in F6P and 3PGA.

(C) Carbon positions 1-C, 2-C, and 3-C of 3PGA map to the five carbon configurations of 3PGA generated from glucose through the OPP pathway. Note that 2-C and 3-C of glucose are rearranged through the OPP pathway whereas 4-C, 5-C, and 6-C of glucose have the same carbon mapping as generated through to the EMP pathway. Glucose-6-phosphate (G6P), 6-phosphogluconate (6PG), ribulose-5-phosphate (RuP), ribose-5-phosphate (R5P), xylulose-5-phosphate (XuP), sedoheptulose-7-phosphate (S7P), erythrose-4-phosphate (E4P), glyceraldehyde-3-phosphate (GAP), fructose-6-phosphate (F6P), 3-phosphoglyceric acid (3PGA). Red outline indicates mapping between 1-C of 3PGA and 3-C or 4-C of G6P. The dashed blue arrows indicate more than 1 reaction.

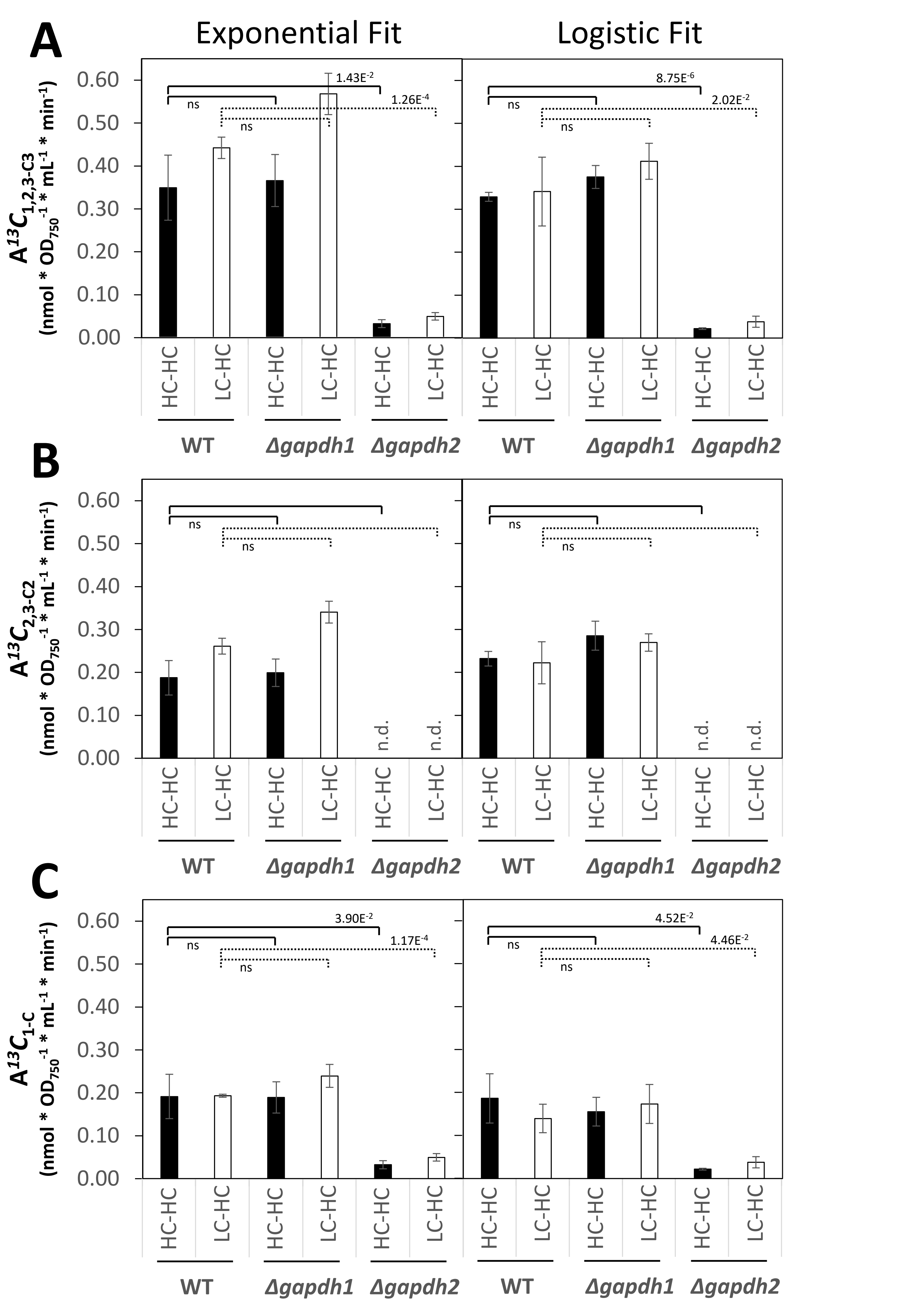
A
B
C
Exponential Fit
Logistic Fit
A13C1,2,3-C3 (nmol * OD750-1 * mL-1 * min-1)
A13C2,3-C2 (nmol * OD750-1 * mL-1 * min-1)
A13C1-C (nmol * OD750-1 * mL-1 * min-1)
0.60
0.50
0.40
0.30
0.20
0.10
0.00
1.43E-2
1.26E-4
8.75E-6
2.02E-2
3.90E-2
1.17E-4
4.52E-2
4.46E-2
ns
n.d.
HC-HC
LC-HC
WT
Δgapdh1
Δgapdh2

Supplementary Figure S3. C assimilation rates (A^{13}C) into positions 1,2,3-C_3, 2,3-C_2, and 1-C of 3PGA from high CO_2 (HC, 5.0 %) and low CO_2 (LC, ambient) pre-acclimated wild-type *Synechocystis sp.* PCC 6803 compared to Δ*gapdh1* and Δ*gapdh2* mutant cells.
Assimilation rates were estimated by the maximum slopes of either logistic (right) or exponential regression functions (left).
(A) $A^{13}C_{1,2,3\text{-}C3}$ (nmol * OD_{750}^{-1} * mL^{-1} * min^{-1}) of 3PGA,
(B) $A^{13}C_{2,3\text{-}C2}$ (nmol * OD_{750}^{-1} * mL^{-1} * min^{-1}) of 3PGA,
(C) $A^{13}C_{1\text{-}C}$ (nmol * OD_{750}^{-1} * mL^{-1} * min^{-1}) of 3PGA.
(Panels on the left) Assimilation rates are estimated as initial rates at t_0 of exponential regression functions applied to data of Supplementary Table S5.
(Panels on the right) Assimilation rates are estimated by midpoint slopes of logistic regression functions applied to the same data (Figure 8A-C).
Cells were probed by a 5.0 % $^{13}CO_2$ (HC) pulse to generate either LC-HC, non-steady state, or HC-HC, steady state, dynamic labelling time series. Brackets indicate Student´s t-test results, ns non-significant, $P < 0.05$ (cf. Supplementary Table S6); n.d. not detected. Data are means ± standard error, n = 3 biological replicates.

A

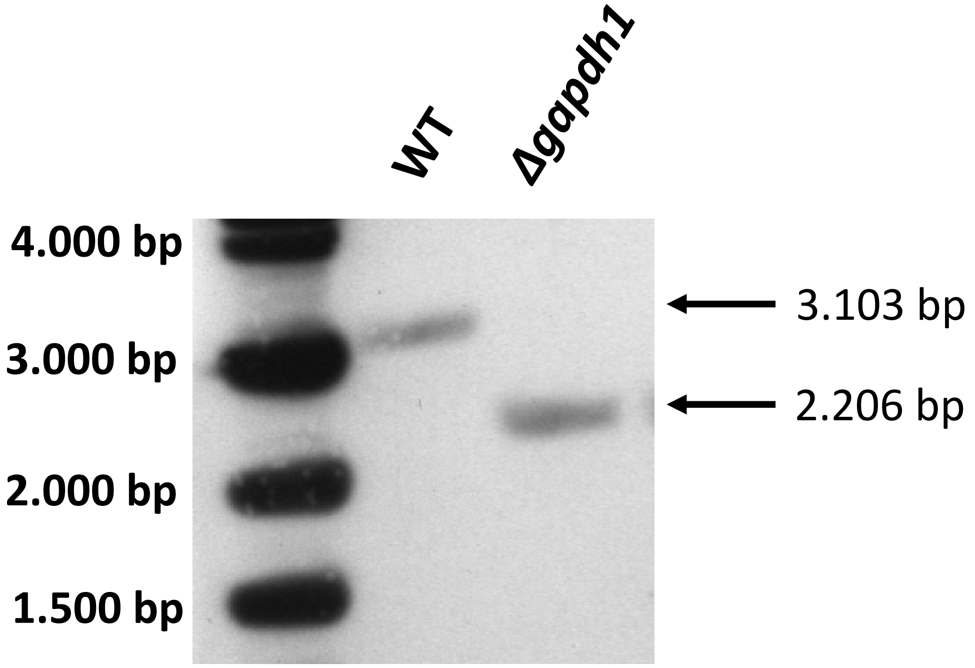

B

Primer	Sequence
Gap1out1	GCGGGTAAGACTATACAGTATCGG
Gap1in1cm	TCAATAATATCGAATTCCTGCAACCAGGACATCCGACTTGCCCTAAC
Gap1in2cm	AGCGGAGGTGCCGCCATCAAGCTTGGCGAGCAAAGACGGTTTGGTCTA
Gap1out2	TACCATGACCGCCGATCAACTGTTG

Supplementary Figure S4. Molecular characterization of the *Δgapdh1* deletion mutant.
(A) Southern blot analysis of wildtype (WT) and *Δgapdh1* (*slr0884*) verify the completed segregation of the *Δgapdh1* mutant. A probe of the *gapdh1* gene detected a fragment with the size of 3,103 bp in the wildtype (WT) and of 2,206 bp in the *Δgapdh1* mutant as expected. This result confirms that *Δgapdh1 is* segregated and that no wild type genome copies are left.
(B) Primer set for the replacement of *gapdh1* (slr0884) by a chloramphenicol resistance cassette for the construction of *Δgapdh1*.

Supplementary Table S1. Validated *in silico* fragmentation analysis of 3-PGA (4TMS) analyzed by GC-EI(TOF)-MS and GC-APCI(TOF)-MS. Mass features, i.e. molecular ions, *in source* adducts, and fragments, with and without C–C bond cleavage of the TMS-derivatized phosphorylated metabolite are indicated by nominal mass (m/z) and characterized by relative abundance of the mass feature within the respective *in source* mass spectra, (-) not detected. Predicted molecular formula are validated by mass accuracy comparing measured to predicted (measured minus predicted) exact masses from GC-APCI(TOF)-MS experiments. Maximally ^{13}C labelled 3PGA was generated by ≥ 90 min photosynthetic *in vivo* labelling experiments of Synechocystis sp. PCC 6803. GC-EI-(TOF)MS spectra of 3PGA(4TMS) for relative base peak abundance analysis were retrieved from GMD (http://gmd.mpimp-golm.mpg.de/; 3PGA (4TMS) identifier A181003). Proposed adducts and cleavage products are reported in square brackets and further validated by mass accuracy of the predicted mass shifts within GC-APCI-(TOF)MS spectra relative to the molecular ion $[M]^+$. % Base peak abundances from GC-EI(TOF)-MS are averages of n = 23 spectra; % base peak abundances from GC-APCI(TOF)-MS are averages of n = 2 spectra.

Mass Feature [m/z] (nominal mass)	Proposed Adducts and Fragmentations	C-Positions of Metabolite (predicted)	Number of *in vivo* labelled ^{13}C-Atoms	% Base Peak Abundance [GC-EI(TOF)-MS]	% Base Peak Abundance [GC-APCI(TOF)-MS]	Molecular Formula (predicted)	Exact Mono-isotopic Mass (predicted)	Mass Accuracy	Exact Mass of fully ^{13}C-labelled Isotopomer (predicted)	Mass Accuracy	Molecular Formula of Adducts and Losses relative to $[M]^+$	Exact Mass Difference (predicted)	Mass Accuracy
547	$[M+TMS]^+$	1C-2C-3C	3	-	44.0	$C_{18}H_{48}O_7PSi_5^+$	547.1979	-0.0021	550.2079	0.0011	C_3H_9Si	73.0474	-0.0006
475	$[M+H]^+$	1C-2C-3C	3	-	100.0	$C_{15}H_{40}O_7PSi_4^+$	475.1583	-0.0016	478.1684	0.0007	H	1.0078	-0.0001
474	$[M]^+$	1C-2C-3C	3	-	0.7	$C_{15}H_{39}O_7PSi_4^+$	474.1505	-0.0015	477.1606	-0.0029	-		
459	$[M-CH_3]^+$	1C-2C-3C	3	13.1	66.1	$C_{14}H_{36}O_7PSi_4^+$	459.1270	-0.0015	462.1371	0.0007	CH_3	-15.0235	0.0000
403	$[M+H+H_2O-TMSOH]^+$	1C-2C-3C	3	-	1.3	$C_{12}H_{32}O_7PSi_3^+$	403.1188	-0.0010	406.1289	0.0006	C_3H_7Si	-71.0317	0.0005
217	$[M-CH_3-(TMS)_2PO_4H]^+$	1C-2C-3C	3	16.1	2.2	$C_8H_{17}O_3PSi_2^+$	217.0711	-0.0003	220.0811	0.0001	$C_7H_{22}O_4PSi_2$	-257.0794	0.0012
143	$[M-CH_3-(TMS)_2PO_4H-TMSH]^+$	1C-2C-3C	3	7.9	-	$C_5H_7O_3Si^+$	143.0159	-	146.0260	-	-	-	-
431	$[M-CH_3-CO]^+$	2C-3C	2	2.3	3.6	$C_{13}H_{36}O_6PSi_4^+$	431.1321	-0.0012	433.1388	0.0013	C_2H_3O	-43.0184	0.0003
415	$[M-CH_3-CO_2]^+$	2C-3C	2	1.6	-	$C_{13}H_{36}O_5PSi_4^+$	415.1372	-	417.1439	-	-	-	-
357	$[M-TMSCOO]^+$	2C-3C	2	37.3	25.6	$C_{11}H_{30}O_5PSi_3^+$	357.1133	-0.0011	359.1200	0.0005	$C_4H_9O_2Si$	-117.0372	0.0004
341	$[M-CH_3-TMSCOOH]^+$	2C-3C	2	5.4	-	$C_{10}H_{26}O_5PSi_3^+$	341.0820	-	343.0887	-	-	-	-
116	$[M-TMSCOO-(TMS)_2PO_4H]^+$	2C-3C	2	6.5	-	$C_5H_{12}OSi^+$	116.0652	-	118.0719	-	-	-	-
101	$[M-TMSCOO-(TMS)_2PO_4H-CH_3]^+$	2C-3C	2	15.8	-	$C_4H_9OSi^+$	101.0417	-	103.0484	-	-	-	-
387	$[PO_4(TMS)_4]^+$	-	0	19.7	22.3	$C_{12}H_{36}O_4PSi_4^+$	387.1423	-0.0012	387.1423	0.0006	$C_3H_3O_3$	-87.0082	0.0003
315	$[PO_4H(TMS)_3]^+$	-	0	20.6	24.3	$C_9H_{28}O_4PSi_3^+$	315.1028	-0.0009	315.1028	0.0006	$C_6H_{11}O_3Si$	-159.0478	0.0006
299	$[PO_4(TMS)_3-CH_3]^+$	-	0	46.4	9.4	$C_8H_{24}O_4PSi_3^+$	299.0715	-0.0008	299.0715	0.0004	$C_7H_{15}O_3Si$	-175.0791	0.0007
227	$[PO_4H(TMS)Si(CH_3)_2]^+$	-	0	27.6	11.9	$C_5H_{16}O_4PSi_2^+$	227.0319	-0.0005	227.0319	-0.0001	$C_{10}H_{23}O_3Si_2$	-247.1186	0.0010
211	$[PO_4H(TMS)Si(CH_3)_2-CH_4]^+$	-	0	23.5	1.6	$C_4H_{12}O_4PSi_2^+$	211.0006	-0.0004	211.0006	0.0000	$C_{11}H_{27}O_3Si_2$	-263.1499	0.0011
147	$[TMSOSi(CH_3)_2]^+$	-	0	39.6	-	$C_5H_{15}OSi_2^+$	147.0656	-	-	-	-	-	-
133	$[TMSOSiH(CH_3)]^+$	-	0	14.5	-	$C_4H_{13}OSi_2^+$	133.0499	-	-	-	-	-	-
73	$[TMS]^+$	-	0	100.0	-	$C_3H_9Si^+$	73.0468	-	-	-	-	-	-

Supplementary Table S2. NIA-corrected $E^{13}C$ of chemically pure non-labelled 3PGA reference substance compared to $E^{13}C$ of 3PGA from complex extracts of non-labelled *Synechocystis sp.* PCC 6803 or *Microcystis aeruginosa* PCC7806.

Paired analyses were performed by GC-APCI-(TOF)MS and GC-EI-(TOF)MS. NIA-corrected $E^{13}C$ of non-labelled substances with ambient isotope composition is expected to be zero. Deviations of measured $E^{13}C$ from zero can result from analytical instrument bias, interference of mass isotopologue distributions by coeluting compounds of overlapping exact masses (GC-APCI-MS) or nominal masses (GC-EI-MS) or by interfering *in source* mass fragments originating directly from 3PGA. Samples of pure 3PGA spanned the complete ranges of 3.0-500 ng injected in splitless mode (GC-APCI-(TOF)MS) or 3.0-150 ng (GC-EI-(TOF)MS). Amounts of 3PGA from the cyanobacterial extracts were within respective ranges (means ± standard error of replications

Metabolite	Mass Feature [m/z]	Adducts or Fragmentations	C-Positions of Metabolite	Molecular Formula	$E^{13}C$ of non-labelled Substances (NIA-corrected)								
					Reference Substance			*Synechocystis sp.* PCC 6803			*Microcystis aeruginosa* PCC7806		
					AVG	SE	n	AVG	SE	n	AVG	SE	n
					**Determined by GC-APCI-MS**								
3-Phosphoglyceric acid (4TMS)	459	$[M-CH_3]^+$	1C-2C-3C	$C_{14}H_{36}O_7PSi_4^+$	0.0000	0.0000	21	0.0000	0.0000	16	0.0000	0.0000	10
	357	$[M-TMSCOO]^+$	2C-3C	$C_{11}H_{30}O_5PSi_3^+$	0.0003	0.0003	21	0.0001	0.0001	16	0.0000	0.0000	10
	459|357	calculated	1C		-0.0006	0.0006	21	-0.0003	0.0003	16	0.0000	0.0000	10
					**Determined by GC-EI-MS**								
3-Phosphoglyceric acid (4TMS)	459	$[M-CH_3]^+$	1C-2C-3C	$C_{14}H_{36}O_7PSi_4^+$	0.0213	0.0035	15	0.0397	0.0061	16	0.0163	0.0072	6
	357	$[M-TMSCOO]^+$	2C-3C	$C_{11}H_{30}O_5PSi_3^+$	0.0186	0.0036	15	0.0034	0.0010	16	0.0044	0.0017	6
	459|357	calculated	1C		0.0267	0.0082	15	0.1123	0.0183	16	0.0401	0.0223	6

Supplementary Table S3. Accuracy and precision of $E^{13}C$ measurements by GC-APCI-(TOF)MS using certified chemical reference substances.

Chemically pure non-labelled glucose, single positional labelled 1-$^{13}C_1$ to 6-$^{13}C_1$-glucoses, fully labelled $^{13}C_6$-glucose and $^{13}C_6$-sorbitol were analyzed by GC-APCI-(TOF)MS. The glucoses were pure reference substances, $^{13}C_6$-sorbitol was added upon extraction as internal standard to preparations of the primary metabolome from *Synechocystis* . Isotopologue distributions of fragment ions representing the full carbon configuration or 3 to 4 carbon atoms of the labelled substance were selected. Accuracy of $E^{13}C$ measurements was assessed by comparing the average measured $E^{13}C$ to the expected isotopic purity range certified by the substance manufacturers. Precision of determined $E^{13}C$ was calculated as standard deviation across replications.

Reference Substance (Chemically Derivatized)	Purity (Analysis Certificate)	Matrix Complexity	Predicted Fragment Ions	Mass Feature [m/z]	Molecular Formula (predicted)	Exact Monoisotopic Mass (predicted)	C-Positions of Compound Included (predicted)	Number of labelled ^{13}C-atoms	$E^{13}C$ expected range	$E^{13}C$ measured (average ± SD)	n
$^{13}C_6$-Sorbitol (6TMS)	> 99 atom % ^{13}C	reference substance in complex extract	$[M-CH_3]^+$	599	$C_{23}H_{59}O_6Si_6^+$	599.29218	1C-2C-3C-4C-5C-6C	6	0.990 - 1.000	0.9994 ± 0.0021	158
$^{13}C_6$-Sorbitol (6TMS)	> 99 atom % ^{13}C	reference substance in complex extract	[M-C12H31O3Si3-TMSOH]$^+$	217	$C_9H_{21}O_2Si_2^+$	217.10746	1C-2C-3C and 4C-5C-6C	3	0.990 - 1.000	0.9947 ± 0.0034	158
$^{13}C_6$-Sorbitol (6TMS)	> 99 atom % ^{13}C	reference substance in complex extract	[M-C8H21O2Si2-TMSOH]$^+$	319	$C_{13}H_{31}O_3Si_3^+$	319.15755	1C-2C-3C-4C and 3C-4C-5C-6C	4	0.990 - 1.000	0.9945 ± 0.0026	158
Glucose (1MEOX) (5TMS)	ambient	pure reference substance	$[M-CH_3]^+$	554	$C_{21}H_{52}NO_6Si_5^+$	554.26355	1C-2C-3C-4C-5C-6C	6	0.000	0.0000 ± 0.0000	2
Glucose (1MEOX) (5TMS)	ambient	pure reference substance	[M-C6H14NO2Si-TMSOH]$^+$	319	$C_{13}H_{31}O_3Si_3^+$	319.15755	3C-4C-5C-6C	4	0.000	0.0000 ± 0.0000	2
$^{13}C_1$-Glucose (1MEOX) (5TMS)	> 99 atom % $^{13}C_1$	pure reference substances	$[M-CH_3]^+$	554	$C_{21}H_{52}NO_6Si_5^+$	554.26355	1C-2C-3C-4C-5C-6C	1	0.165 - 0.167	0.1667 ± 0.0001	12
$^{13}C_1$-Glucose (1MEOX) (5TMS)	> 99 atom % $^{13}C_1$	pure reference substances	[M-C6H14NO2Si-TMSOH]$^+$	319	$C_{13}H_{31}O_3Si_3^+$	319.15755	3C-4C-5C-6C	1	0.248 - 0.250	0.2462 ± 0.0007	8
$^{13}C_6$-Glucose (1MEOX) (5TMS)	> 99 atom % ^{13}C	pure reference substance	$[M-CH_3]^+$	554	$C_{21}H_{52}NO_6Si_5^+$	554.26355	1C-2C-3C-4C-5C-6C	6	0.990 - 1.000	0.9945 ± 0.0007	2
$^{13}C_6$-Glucose (1MEOX) (5TMS)	> 99 atom % ^{13}C	pure reference substance	[M-C6H14NO2Si-TMSOH]$^+$	319	$C_{13}H_{31}O_3Si_3^+$	319.15755	3C-4C-5C-6C	4	0.990 - 1.000	0.9948 ± 0.0001	2

Supplementary Table S4. Relative standard deviations (RSD) of 3PGA quantifications by GC-EI-MS using either sums of NIA-corrected isotopologue abundances or monoisotopic mass fragments that did not incorporate ^{13}C.

3PGA was quantified in complex samples (n = 168) from dynamic $^{13}CO_2$ labelling experiments of *Synechocystis* cells. The quantitative calibration was performed by co-analysis of dilution series of non-labelled 3PGA reference substance. Calibration samples and complex samples were internally standardized by $^{13}C_6$-sorbitol. 3PGA was quantified separately using the fragment ions 459, 357, 299, and 315. Fragment ions 357 and 459 were quantified through the NIA-corrected sums of isotopologue distributions to account for differential ^{13}C labeling across samples. Mass fragments 299 and 315 did not contain labelled carbon atoms and were analyzed through the abundance of their monoisotopic mass to charge ratios. Pearson´s correlation coefficients (r) assuming linear correlation between separate quantifications using each of the 4 fragment ions are reported. RSDs were calculated across the steady-state HC-HC time series of wild type and of 2 mutants separately assuming constant 3PGA concentrations. These RSDs were compared to identical RSD calculations from timeseries of the LC-HC state transitions. Analyses were performed using relative 3PGA abundances with arbitrary units (top) and after quantifying 3PGA concentrations as nmol * OD_{750}^{-1} * mL^{-1} (bottom). Note that fragment ions had different relative mass spectral base peak abundance, fragment ion 459 was at 13.1 %, 315 at 20.6 %, 357 at 37.3 %, and 299 at 46.4 % (rf. Supplementary Table S1).

3PGA	**Pearson´s correlation coefficient (r)**				**Relative standard deviation (%)**			**Relative standard deviation (%)**		
Relative Abundance (arbitrary units)	fragment 459	fragment 357	fragment 299	fragment 315	Δ*gapdh1* (HC->HC)	Δ*gapdh2* (HC->HC)	WT (HC->HC)	Δ*gapdh1* (LC->HC)	Δ*gapdh2* (LC->HC)	WT (LC->HC)
fragment 459					40.06	49.20	30.00	32.64	44.53	42.46
fragment 357	0.996				35.67	42.27	26.44	27.29	38.34	39.55
fragment 299	0.997	0.999			34.59	40.01	25.25	26.23	36.50	36.95
fragment 315	0.996	0.999	1.000		34.26	40.12	25.47	26.17	37.72	38.11
Concentration (nmol * OD_{750}^{-1} * mL^{-1}) determined by	fragment 459	fragment 357	fragment 299	fragment 315	Δ*gapdh1* (HC->HC)	Δ*gapdh2* (HC->HC)	WT (HC->HC)	Δ*gapdh1* (LC->HC)	Δ*gapdh2* (LC->HC)	WT (LC->HC)
fragment 459					10.54	13.57	13.12	17.24	14.85	22.47
fragment 357	0.982				9.45	13.19	12.36	12.99	11.25	20.58
fragment 299	0.962	0.994			9.58	13.47	12.27	11.78	9.78	18.70
fragment 315	0.925	0.977	0.993		9.94	13.64	12.24	10.30	8.86	17.76

Supplementary Table S5. 3PGA concentration and molar carbon assimilation analysis into positions 1-C, 2,3-C_2 and 1,2,3-C_3 of 3PGA of high CO_2 (HC, 5.0 %) and low CO_2 (LC, ambient) pre-acclimated wild type, Δ*gapdh1* and Δ*gapdh2* mutant cells of *Synechocystis sp.* PCC 6803.

Cells were probed by a 5.0 % $^{13}CO_2$ (HC) pulse to generate non-steady state LC-HC dynamic labelling series or HC-HC steady state labelling data. 3PGA concentrations, C_{3PGA} (nmol * OD_{750}^{-1} * mL^{-1}), were quantified by GC-EI-MS technology. Carbon assimilatio, $C^{13}C$ (nmol * OD_{750}^{-1} * mL^{-1}), was calculated from C_{3PGA} using $E^{13}C$ data of paired GC-APCI-MS analyses. Three independent experiments of HC- and LC-pre-acclimated cultures were performed in photobioreactors (columns BH-CR). Data are averaged with standard error calculations (columns C-Z). Fold-Changes and significance of differences between HC and LC cells are listed (columns AA-AL) using the heteroscedastic, two-tailed Student´s t-test, $P \leq 0.05$ (light green, $P \leq 0.01$ (green), and $P \leq 0.001$ (dark green). Fold changes (FC) are color coded, unchanged FC = 1 (yellow), FC ≤ 0.2 (blue), FC ≥ 5 (red). Note that *Δgapdh2* mutant cells are not viable under photoautotrophic conditions. Δ*Gapdh2* mutant cells were pre-cultivated in the presence of 10 mM non-labelled glucose in BG11 medium. The $^{13}CO_2$ (HC) pulse was in all cases in the absence of external glucose. The table contains sections of the concentration of 3PGA, molar assimilation of carbon into 1,2,3-C_3, 2,3-C_2, and 1-C of 3PGA, and of the rel. $E^{13}C_{2,3\text{-}C2/1\text{-}C}$ (%). $E^{13}C_{2,3\text{-}C2}$ of Δ*gapdh2* mutant cells was equal to non-labelled ambient 3PGA.

Parameter	Genotype	HC->HC 0 (min) AVG	5 (min) AVG	10 (min) AVG	15 (min) AVG	30 (min) AVG	60 (min) AVG	LC->HC 0 (min) AVG	5 (min) AVG	10 (min) AVG	15 (min) AVG	30 (min) AVG	60 (min) AVG	HC->HC 0 (min) SE	5 (min) SE	10 (min) SE	15 (min) SE	30 (min) SE	60 (min) SE	LC->HC 0 (min) SE	5 (min) SE	10 (min) SE	15 (min) SE	30 (min) SE	60 (min) SE	ratio (LC vs. HC) 00 min FC	ratio (LC vs. HC) 05 min FC	ratio (LC vs. HC) 10 min FC	ratio (LC vs. HC) 15 min FC	ratio (LC vs. HC) 30 min FC	ratio (LC vs. HC) 60 min FC	t-test (LC vs. HC) 00 min *P*	t-test (LC vs. HC) 05 min *P*	t-test (LC vs. HC) 10 min *P*	t-test (LC vs. HC) 15 min *P*	t-test (LC vs. HC) 30 min *P*	t-test (LC vs. HC) 60 min *P*
**Concentration of 3PGA**																																					
C_{3PGA} (nmol * OD_{750}^{-1} * mL^{-1})	WT	1.396	1.249	1.322	1.406	1.270	1.253	1.863	1.042	1.121	1.296	1.371	1.366	0.070	0.202	0.042	0.119	0.107	0.058	0.087	0.080	0.130	0.024	0.013	0.146	1.33	0.83	0.85	0.92	1.08	1.09	0.015	0.421	0.259	0.457	0.447	0.531
C_{3PGA} (nmol * OD_{750}^{-1} * mL^{-1})	*Δgapdh1*	1.190	1.415	1.441	1.601	1.431	1.421	1.746	1.252	1.437	1.682	1.794	1.748	0.061	0.008	0.048	0.097	0.023	0.068	0.017	0.122	0.087	0.062	0.086	0.077	1.47	0.88	1.00	1.05	1.25	1.23	0.008	0.312	0.972	0.526	0.043	0.034
C_{3PGA} (nmol * OD_{750}^{-1} * mL^{-1})	*Δgapdh2*	0.872	0.983	0.899	0.992	1.035	1.163	0.816	0.719	0.668	0.686	0.712	0.689	0.069	0.057	0.076	0.050	0.083	0.055	0.091	0.054	0.044	0.061	0.005	0.025	0.94	0.73	0.74	0.69	0.69	0.59	0.650	0.028	0.074	0.019	0.059	0.006
Fold Change (FC)	*Δgapdh1* vs WT	0.85	1.13	1.09	1.14	1.13	1.13	0.94	1.20	1.28	1.30	1.31	1.28																								
Fold Change (FC)	*Δgapdh2* vs WT	0.62	0.79	0.68	0.71	0.81	0.93	0.44	0.69	0.60	0.53	0.52	0.50																								
t-test (*P*)	*Δgapdh1* vs WT	0.092	0.498	0.135	0.276	0.269	0.134	0.312	0.232	0.124	0.015	0.036	0.103																								
t-test (*P*)	*Δgapdh2* vs WT	0.006	0.318	0.015	0.058	0.162	0.326	0.001	0.034	0.061	0.005	0.000	0.040																								
**Assimilation of Carbon into 1,2,3-C_3 of 3PGA**																																					
C^{13} $C_{1,2,3\text{-}C3}$ (nmol * OD_{750}^{-1} * mL^{-1})	WT	0.000	0.928	2.280	3.049	3.205	3.426	0.000	1.595	2.631	3.404	3.825	3.934	0.000	0.371	0.152	0.499	0.410	0.207	0.000	0.178	0.344	0.084	0.092	0.464		1.72	1.15	1.12	1.19	1.15		0.207	0.424	0.552	0.267	0.397
C^{13} $C_{1,2,3\text{-}C3}$ (nmol * OD_{750}^{-1} * mL^{-1})	*Δgapdh1*	0.000	0.854	2.396	3.455	3.563	3.884	0.000	1.957	3.382	4.463	5.100	5.125	0.000	0.279	0.321	0.358	0.136	0.209	0.000	0.159	0.215	0.150	0.221	0.236		2.29	1.41	1.29	1.43	1.32		0.038	0.072	0.090	0.007	0.018
C^{13} $C_{1,2,3\text{-}C3}$ (nmol * OD_{750}^{-1} * mL^{-1})	*Δgapdh2*	0.000	0.153	0.231	0.310	0.386	0.493	0.000	0.177	0.249	0.280	0.330	0.315	0.000	0.068	0.042	0.051	0.056	0.058	0.000	0.023	0.016	0.029	0.011	0.016		1.16	1.08	0.90	0.86	0.64		0.757	0.724	0.640	0.428	0.082
Fold Change (FC)	*Δgapdh1* vs WT		0.92	1.05	1.13	1.11	1.13		1.23	1.29	1.31	1.33	1.30																								
Fold Change (FC)	*Δgapdh2* vs WT		0.16	0.10	0.10	0.12	0.14		0.11	0.09	0.08	0.09	0.08																								
t-test (*P*)	*Δgapdh1* vs WT		0.881	0.766	0.549	0.480	0.194		0.204	0.151	0.007	0.017	0.107																								
t-test (*P*)	*Δgapdh2* vs WT		0.168	0.003	0.030	0.019	0.003		0.014	0.020	0.000	0.001	0.016																								
**Assimilation of Carbon into 2,3-C_2 of 3PGA**																																					
C^{13} $C_{2,3\text{-}C2}$ (nmol * OD_{750}^{-1} * mL^{-1})	WT	0.000	0.338	1.326	1.924	2.060	2.228	0.000	0.883	1.632	2.205	2.513	2.589	0.000	0.181	0.101	0.344	0.279	0.143	0.000	0.096	0.225	0.063	0.081	0.312		2.62	1.23	1.15	1.22	1.16		0.075	0.310	0.501	0.242	0.375
C^{13} $C_{2,3\text{-}C2}$ (nmol * OD_{750}^{-1} * mL^{-1})	*Δgapdh1*	0.000	0.265	1.390	2.185	2.295	2.524	0.000	1.090	2.141	2.910	3.374	3.400	0.000	0.123	0.232	0.241	0.092	0.127	0.000	0.091	0.143	0.104	0.145	0.155		4.12	1.54	1.33	1.47	1.35		0.007	0.063	0.078	0.006	0.013
C^{13} $C_{2,3\text{-}C2}$ (nmol * OD_{750}^{-1} * mL^{-1})	*Δgapdh2*	0.000	0.000	0.001	0.000	0.000	0.000	0.000	0.000	0.000	0.000	0.000	0.000	0.000	0.000	0.001	0.000	0.000	0.000	0.000	0.000	0.000	0.000	0.000	0.000												
Fold Change (FC)	*Δgapdh1* vs WT		0.78	1.05	1.14	1.11	1.13		1.23	1.31	1.32	1.34	1.31																								
Fold Change (FC)	*Δgapdh2* vs WT		0.00	0.00	0.00	0.00	0.00		0.00	0.00	0.00	0.00	0.00																								
t-test (*P*)	*Δgapdh1* vs WT		0.757	0.820	0.571	0.494	0.198		0.193	0.142	0.008	0.012	0.104																								
t-test (*P*)	*Δgapdh2* vs WT		0.165	0.027	0.012	0.002	0.003		0.007	0.004	0.001	0.002	0.002																								
**Assimilation of Carbon into 1-C of 3PGA**																																					
C^{13} $C_{1\text{-}C}$ (nmol * OD_{750}^{-1} * mL^{-1})	WT	0.000	0.590	0.953	1.125	1.145	1.198	0.000	0.712	0.999	1.199	1.313	1.345	0.000	0.190	0.053	0.155	0.131	0.063	0.000	0.082	0.121	0.025	0.011	0.153		1.21	1.05	1.07	1.15	1.12		0.603	0.756	0.683	0.329	0.448
C^{13} $C_{1\text{-}C}$ (nmol * OD_{750}^{-1} * mL^{-1})	*Δgapdh1*	0.000	0.589	1.006	1.269	1.268	1.360	0.000	0.867	1.241	1.553	1.726	1.725	0.000	0.156	0.090	0.117	0.044	0.082	0.000	0.068	0.073	0.046	0.076	0.081		1.47	1.23	1.22	1.36	1.27		0.210	0.116	0.123	0.012	0.034
C^{13} $C_{1\text{-}C}$ (nmol * OD_{750}^{-1} * mL^{-1})	*Δgapdh2*	0.000	0.153	0.230	0.310	0.386	0.493	0.000	0.177	0.249	0.280	0.330	0.315	0.000	0.068	0.042	0.051	0.056	0.058	0.000	0.023	0.016	0.029	0.011	0.016		1.16	1.08	0.90	0.86	0.64		0.757	0.716	0.640	0.428	0.082
Fold Change (FC)	*Δgapdh1* vs WT		1.00	1.06	1.13	1.11	1.13		1.22	1.24	1.29	1.31	1.28																								
Fold Change (FC)	*Δgapdh2* vs WT		0.26	0.24	0.28	0.34	0.41		0.25	0.25	0.23	0.25	0.23																								
t-test (*P*)	*Δgapdh1* vs WT		0.997	0.644	0.502	0.451	0.197		0.222	0.178	0.006	0.030	0.114																								
t-test (*P*)	*Δgapdh2* vs WT		0.136	0.001	0.025	0.017	0.001		0.017	0.024	0.000	0.000	0.020																								
**Rel. E^{13} $C_{2,3\text{-}C2/1\text{-}C\ (3PGA)}$ %**																																					
Rel. E^{13} $C_{2,3\text{-}C2/1\text{-}C\ (3PGA)}$ %	WT	0.0	24.6	69.4	84.6	89.5	92.8	0.0	62.2	81.5	92.0	95.7	96.1	0.0	6.6	2.4	3.3	2.0	1.1	0.0	1.0	3.0	1.7	2.3	1.5		2.53	1.17	1.09	1.07	1.04		0.027	0.036	0.140	0.115	0.158
Rel. E^{13} $C_{2,3\text{-}C2/1\text{-}C\ (3PGA)}$ %	*Δgapdh1*	0.0	18.6	68.2	85.8	90.4	92.9	0.0	62.9	86.2	93.7	97.8	98.6	0.0	7.4	5.2	1.9	0.6	0.9	0.0	0.9	0.7	0.5	0.2	0.2		3.39	1.26	1.09	1.08	1.06		0.025	0.072	0.042	0.003	0.023
Rel. E^{13} $C_{2,3\text{-}C2/1\text{-}C\ (3PGA)}$ %	*Δgapdh2*	0.0	0.0	0.1	0.0	0.0	0.0	0.0	0.0	0.0	0.0	0.0	0.0	0.0	0.0	0.1	0.0	0.0	0.0	0.0	0.0	0.0	0.0	0.0	0.0												
Fold Change (FC)	*Δgapdh1* vs WT		0.75	0.98	1.01	1.01	1.00		1.01	1.06	1.02	1.02	1.03																								
Fold Change (FC)	*Δgapdh2* vs WT		0.00	0.00	0.00	0.00	0.00		0.00	0.00	0.00	0.00	0.00																								
t-test (*P*)	*Δgapdh1* vs WT		0.574	0.840	0.780	0.714	0.966		0.622	0.245	0.427	0.453	0.237																								
t-test (*P*)	*Δgapdh2* vs WT		0.128	0.006	0.000	0.000	0.000		0.000	0.000	0.000	0.000	0.000																								

Supplementary Table S6. Assimilation rates of ^{13}C into 1,2,3-C_3, 2,3-C_2, and 1-C of 3PGA, estimated by exponential or logistic sigmoidal fitting of dynamic molar $^{13}CO_2$ assimilation kinetics of high CO_2 (HC, 5.0 %) and low CO_2 (LC, ambient) pre-acclimated wild type, Δ*gapdh1* and Δ*gapdh2* mutant cells of *Synechocystis sp.* PCC 6803. Cells were probed by a 5.0 % $^{13}CO_2$ (HC) pulse to generate non-steady state LC-HC dynamic labelling series or HC-HC steady state labelling data. 3PGA concentrations, C_{3PGA} (nmol * $OD750^{-1}$ * mL^{-1}), were quantified by GC-EI-(TOF)MS technology. Carbon assimilation, $C^{13}C$ (nmol * $OD750^{-1}$ * mL^{-1}), was calculated from C_{3PGA} using $E^{13}C$ data of paired GC-APCI-(TOF)MS analyses. Three independent experiments of HC- and LC-pre-acclimated cultures were performed in photobioreactors (Supplemental Table 5). Initial rates at t_0 from exponential fittings or midpoint slopes (nmol ^{13}C * $OD750^{-1}$ * mL^{-1} * min^{-1}) and times (min) from logistic fitting are averaged with standard error calculations and two-tailed Student´s t-tests of differences between LC-HC and HC-HC experiments. Alternatively, each mutant genotype is compared against the WT for changes at LC or HC pre-acclimation conditions. Test results of logistic sigmoidal fits are included (columns C-E). Note that Δ*gapdh2* mutant cells are not viable under photoautotrophic conditions. Δ*Gapdh2* mutant cells were pre-cultivated in the presence of 10 mM non-labelled glucose in BG11 medium. The $^{13}CO_2$ (HC) pulse was in all cases in the absence of external glucose. Significant (P <0.05) fold-changes are highlighted by red font.

Genotype	Experiment	exponential fitting: initial slope at t_0 (nmol ^{13}C * $OD750^{-1}$ * mL^{-1} * min^{-1})	logistic fitting: slope at midPoint (nmol ^{13}C * $OD750^{-1}$ * mL^{-1} * min^{-1})	midPoint (min)	log_likelihood	isThisaFit	model
**Assimilation of Carbon into 1,2,3-C_3 of 3PGA**							
WT	HC-HC	0.23	0.31	8.51	6.85	TRUE	sigmoidal
	HC-HC	0.32	0.35	7.54	11.62	TRUE	sigmoidal
	HC-HC	0.49	0.33	7.16	7.37	TRUE	sigmoidal
WT	LC-HC	0.43	0.28	7.66	6.87	TRUE	sigmoidal
	LC-HC	0.41	0.24	7.96	5.82	TRUE	sigmoidal
	LC-HC	0.49	0.50	5.98	11.30	TRUE	sigmoidal
Δ*gapdh1*	HC-HC	0.25	0.33	9.61	10.57	TRUE	sigmoidal
	HC-HC	0.46	0.43	6.88	13.04	TRUE	sigmoidal
	HC-HC	0.38	0.37	8.75	8.34	TRUE	sigmoidal
Δ*gapdh1*	LC-HC	0.59	0.37	7.67	8.35	TRUE	sigmoidal
	LC-HC	0.64	0.50	5.93	10.52	TRUE	sigmoidal
	LC-HC	0.48	0.37	8.62	10.21	TRUE	sigmoidal
Δ*gapdh2*	HC-HC	0.02	0.02	12.15	6.85	TRUE	sigmoidal
	HC-HC	0.03	0.02	9.92	6.34	TRUE	sigmoidal
	HC-HC	0.05	0.03	8.45	5.37	TRUE	sigmoidal
Δ*gapdh2*	LC-HC	0.04	0.02	5.70	5.74	TRUE	sigmoidal
	LC-HC	0.07	0.06	4.19	8.60	TRUE	sigmoidal
	LC-HC	0.04	0.03	5.98	7.12	TRUE	sigmoidal
**Assimilation of Carbon into 2,3-C_2 of 3PGA**							
WT	HC-HC	0.12	0.20	9.53	6.20	TRUE	sigmoidal
	HC-HC	0.18	0.26	8.39	10.39	TRUE	sigmoidal
	HC-HC	0.26	0.24	8.66	8.99	TRUE	sigmoidal
WT	LC-HC	0.25	0.18	8.60	7.67	TRUE	sigmoidal
	LC-HC	0.24	0.16	8.90	6.95	TRUE	sigmoidal
	LC-HC	0.30	0.32	6.37	11.50	TRUE	sigmoidal
Δ*gapdh1*	HC-HC	0.14	0.22	10.60	9.19	TRUE	sigmoidal
	HC-HC	0.25	0.32	7.94	13.54	TRUE	sigmoidal
	HC-HC	0.21	0.32	9.93	9.63	TRUE	sigmoidal
Δ*gapdh1*	LC-HC	0.35	0.25	8.34	9.61	TRUE	sigmoidal
	LC-HC	0.38	0.31	6.67	11.07	TRUE	sigmoidal
	LC-HC	0.29	0.25	9.18	11.22	TRUE	sigmoidal
Δ*gapdh2*	HC-HC	n.d.	n.d.	n.d.	n.d.	n.d.	n.d.
	HC-HC	n.d.	n.d.	n.d.	n.d.	n.d.	n.d.
	HC-HC	n.d.	n.d.	n.d.	n.d.	n.d.	n.d.
Δ*gapdh2*	LC-HC	n.d.	n.d.	n.d.	n.d.	n.d.	n.d.
	LC-HC	n.d.	n.d.	n.d.	n.d.	n.d.	n.d.
	LC-HC	n.d.	n.d.	n.d.	n.d.	n.d.	n.d.
**Assimilation of Carbon into 1-C of 3PGA**							
WT	HC-HC	0.12	0.12	6.53	7.72	TRUE	sigmoidal
	HC-HC	0.16	0.14	5.67	11.48	TRUE	sigmoidal
	HC-HC	0.29	0.30	3.91	6.34	TRUE	sigmoidal
WT	LC-HC	0.19	0.13	5.31	5.76	TRUE	sigmoidal
	LC-HC	0.19	0.09	5.94	4.12	TRUE	sigmoidal
	LC-HC	0.20	0.20	5.27	10.97	TRUE	sigmoidal
Δ*gapdh1*	HC-HC	0.12	0.12	7.73	12.26	TRUE	sigmoidal
	HC-HC	0.25	0.22	4.54	10.46	TRUE	sigmoidal
	HC-HC	0.20	0.12	6.11	6.59	TRUE	sigmoidal
Δ*gapdh1*	LC-HC	0.24	0.13	6.19	6.48	TRUE	sigmoidal
	LC-HC	0.28	0.26	4.56	10.63	TRUE	sigmoidal
	LC-HC	0.19	0.12	7.43	8.42	TRUE	sigmoidal
Δ*gapdh2*	HC-HC	0.02	0.02	12.15	6.85	TRUE	sigmoidal
	HC-HC	0.03	0.02	9.98	6.35	TRUE	sigmoidal
	HC-HC	0.05	0.03	8.45	5.37	TRUE	sigmoidal
Δ*gapdh2*	LC-HC	0.04	0.02	5.70	5.74	TRUE	sigmoidal
	LC-HC	0.07	0.06	4.19	8.60	TRUE	sigmoidal
	LC-HC	0.04	0.03	5.98	7.12	TRUE	sigmoidal

Genotype	Experiment	exponential fitting initial slope at t_0 (nmol ^{13}C * OD_{750}^{-1} * mL^{-1} * min^{-1})							logistic fitting slope at midPoint (nmol ^{13}C * OD_{750}^{-1} * mL^{-1} * min^{-1})												logistic fitting midPoint (min)											
		AVG	SD	SE	LC-HC vs HC-HC fold-change	LC-HC vs HC-HC *P* (t-test)	mutant vs WT fold-change	mutant vs WT *P* (t-test)	AVG	SD	SE	LC-HC vs HC-HC fold-change	LC-HC vs HC-HC *P* (t-test)	mutant vs WT fold-change	mutant vs WT *P* (t-test)	AVG [LC&HC]	SD [LC&HC]	SE [LC&HC]	mutant vs WT fold-change	mutant vs WT *P* (t-test)	AVG	SD	SE	LC-HC vs HC-HC fold-change	LC-HC vs HC-HC *P* (t-test)	mutant vs WT fold-change	mutant vs WT *P* (t-test)	AVG [LC&HC]	SD [LC&HC]	SE [LC&HC]	mutant vs WT fold-change	mutant vs WT *P* (t-test)
**Assimilation of Carbon into 1,2,3-C_3 of 3PGA**																																
WT	HC-HC	**0.35**	0.13	0.08					**0.33**	0.02	0.01										**7.7**	0.7	0.4									
WT	LC-HC	**0.44**	0.04	0.03	1.3	3.1E-01			**0.34**	0.14	0.08	1.0	8.8E-01			0.34	0.09	0.04			**7.2**	1.1	0.6	0.9	5.1E-01			7.5	0.9	0.4		
Δ*gapdh1*	HC-HC	**0.37**	0.10	0.06			1.0	8.7E-01	**0.38**	0.05	0.03			1.1	1.8E-01						**8.4**	1.4	0.8			1.1	3.7E-01					
Δ*gapdh1*	LC-HC	**0.57**	0.08	0.05	1.6	6.0E-02	1.3	8.2E-02	**0.41**	0.07	0.04	1.1	5.0E-01	1.2	4.8E-01	0.39	0.06	0.02	1.2	2.1E-01	**7.4**	1.4	0.8	0.9	4.2E-01	1.0	4.0E-01	7.9	1.4	0.6	1.1	5.1E-01
Δ*gapdh2*	HC-HC	**0.03**	0.02	0.01			0.1	1.4E-02	**0.02**	0.00	0.00			0.1	8.7E-06						**10.2**	1.9	1.1			1.3	2.0E-01					
Δ*gapdh2*	LC-HC	**0.05**	0.02	0.01	1.5	2.6E-01	0.1	1.3E-04	**0.04**	0.02	0.01	1.7	3.0E-01	0.1	2.0E-02	0.03	0.02	0.01	0.1	8.8E-06	**5.3**	1.0	0.6	0.5	1.6E-02	0.7	6.8E-01	7.7	3.0	1.2	1.0	8.4E-01
**Assimilation of Carbon into 2,3-C_2 of 3PGA**																																
WT	HC-HC	**0.19**	0.07	0.04					**0.23**	0.03	0.02										**8.9**	0.6	0.3									
WT	LC-HC	**0.26**	0.03	0.02	1.4	1.7E-01			**0.22**	0.08	0.05	1.0	8.6E-01			0.23	0.06	0.02			**8.0**	1.4	0.8	0.9	3.6E-01			8.4	1.1	0.4		
Δ*gapdh1*	HC-HC	**0.20**	0.06	0.03			1.1	8.3E-01	**0.29**	0.06	0.03			1.2	2.3E-01						**9.5**	1.4	0.8			1.1	2.9E-01					
Δ*gapdh1*	LC-HC	**0.34**	0.04	0.03	1.7	2.6E-02	1.3	6.5E-02	**0.27**	0.03	0.02	0.9	7.1E-01	1.2	4.2E-01	0.28	0.04	0.02	1.2	1.2E-01	**8.1**	1.3	0.7	0.8	2.6E-01	1.0	2.7E-01	8.8	1.4	0.6	1.0	6.2E-01
**Assimilation of Carbon into 1-C of 3PGA**																																
WT	HC-HC	**0.19**	0.09	0.05					**0.19**	0.10	0.06										**5.4**	1.3	0.8									
WT	LC-HC	**0.19**	0.01	0.00	1.0	9.7E-01			**0.14**	0.06	0.03	0.7	5.2E-01			0.16	0.08	0.03			**5.5**	0.4	0.2	1.0	8.7E-01			5.4	0.9	0.4		
Δ*gapdh1*	HC-HC	**0.19**	0.06	0.04			1.0	9.8E-01	**0.16**	0.06	0.03			0.8	6.6E-01						**6.1**	1.6	0.9			1.1	6.1E-01					
Δ*gapdh1*	LC-HC	**0.24**	0.05	0.03	1.3	3.3E-01	1.2	1.6E-01	**0.17**	0.08	0.05	1.1	7.7E-01	1.2	5.8E-01	0.16	0.06	0.03	1.0	9.7E-01	**6.1**	1.4	0.8	1.0	9.6E-01	1.1	5.5E-01	6.1	1.4	0.6	1.1	3.4E-01
Δ*gapdh2*	HC-HC	**0.03**	0.02	0.01			0.2	3.9E-02	**0.02**	0.00	0.00			0.1	4.5E-02						**10.2**	1.9	1.1			1.9	2.2E-01					
Δ*gapdh2*	LC-HC	**0.05**	0.02	0.01	1.5	2.6E-01	0.3	1.2E-04	**0.04**	0.02	0.01	1.7	3.0E-01	0.3	4.5E-02	0.03	0.02	0.01	0.2	1.9E-03	**5.3**	1.0	0.6	0.5	1.5E-02	1.0	9.3E-01	7.7	3.0	1.2	1.4	1.0E-01
